# Excess mortality in young cancer survivors compared with the general population in Italy: a retrospective study from the Italian population-based cohort of adolescents and young adult cancer survivors

**DOI:** 10.3389/fonc.2025.1580953

**Published:** 2025-07-30

**Authors:** Paolo Giorgi Rossi, Francesco Marinelli, Pamela Mancuso, Lucia Mangone, Massimo Vicentini, Isabella Bisceglia, Alice Bernasconi, Laura Botta, Annalisa Trama, Ada Working Group

**Affiliations:** ^1^ Epidemiology Unit, AUSL-IRCCS di Reggio Emilia, Reggio Emilia, Italy; ^2^ Evaluative Epidemiology Unit, Department of Research, Fondazione IRCCS Istituto Nazionale dei Tumori, Milan, Italy

**Keywords:** survivors, adolescent and young adults, cancer, cohort study, mortality

## Abstract

**Background:**

Adolescents and young adults (AYA) cancer survivors experience increased morbidity and mortality from second cancers, cardiovascular, infectious, kidney, and other chronic diseases. We aim to calculate all-causes cancer and non-cancer excess mortality of young cancer survivors compared to the general population.

**Methods:**

The AYA cohort includes cancer patients diagnosed between 1976 and 2013 and alive at 5 years after diagnosis in 30 population-based Cancer Registries and followed up until 31 December 2019. The standardised mortality ratios (SMRs) and absolute excess risks (AERs) per 100,000 for person-years were calculated.

**Results:**

58,387 5-year survivors were followed up for 427,130 person-years; the median follow-up time was 5.7 years beyond the 5^th^ year after diagnosis. During this time, 4,194 (7.2%) had died by the end of 2019, and only 1.6% were lost to follow-up. Compared with the general population, AYA survivors had higher mortality, overall, the SMR for all-cause mortality was 7.0 (95%CI: 6.8-7.2). The excess of mortality was higher in the first period after diagnosis (5–10 years), SMR 12.8 (95%CI 12.3-13.3), then it decreased, reaching an SMR of 2.2 (95%CI 1.6-3.2) after 30 years.

**Conclusions:**

The excess mortality is mostly due to the malignancy of the primary tumour, but an about 2-fold excess of mortality is also appreciable for non-cancer causes. Young adult cancer survivors face a sevenfold increase in all-cause mortality compared to the general population, with a notable rise in both cancer-related and non-cancer deaths. Thirty years post-diagnosis, the excess risk from cancer and non-cancer causes becomes nearly equal.

## 1 Introduction

Cancers in adolescents and young adults (15–39 years at cancer diagnosis) are rare. About 5% of all new cases occurs among ages 15 to 39 (1, 2). More than 80% of AYAs diagnosed with cancer will survive their cancer for 5 years after diagnosis (3, 4). Nevertheless, cancer is the most common cause of disease-related death in adolescents and young adults (AYAs) in high-income countries (HICs) (5, 6).

The most common cancer type among AYAs in Europe is female breast cancer, with an age-adjusted rate of 24.4 new cases per 100,000 female AYAs, followed by testicular cancer (14.5 per 100,000 male AYAs), cervix (8.5, per 100,000 female AYAs), thyroid cancer (8.4 per 100,000 AYAs), and melanoma (8.0 per 100,000 AYAs) (2).

In the last 50 years, the survival of patients having cancers in their adolescence or early adulthood has improved in high-income countries thanks to earlier diagnosis, better therapies, and better organization of health care (7).

In AYAs, the incidence trend for all cancers increased by 1% per year (APC 1[95%CI 0.9-1.2]) from 1998 to 2006 and stabilized until 2019. Nevertheless, this overall increasing trend results from different trends of specific cancers, some of which had a stable incidence, some increased, and some decreased (8).

The high survival allowed large cohorts of AYA cancer patients to enter at older ages. However, AYA cancer survivors experience multiple long-term effects of cancer, which begin after completion of treatment, and significantly impact their quality of life and increase their risk of death (7). There are marked differences in risks and types of Subsequent Malignant Neoplasms (SMNs) among survivors, with solid tumours being notably prevalent (9). Cause-specific mortality (10) and hospitalization patterns indicate higher rates of complications from infections, respiratory conditions, and diseases related to the blood and hematopoietic system, particularly among leukaemia survivors (9).

Long-term effects, which begin during treatment and persist thereafter, may be due to several risk factors interacting together, including cancer treatment, lifestyle, genetic susceptibility, and environmental and occupational exposure (11, 12). Some of these factors are country-specific and so is their impact on cancer survivors. Thus, although cancers in AYAs are rare, country-specific research is also needed. Studies on long-term effects in AYA cancer survivors are available in different western countries (10, 11, 13), but are very limited in Italy (9, 14).

Thus, we leveraged the Italian AYA cancer survivor’s cohort (15) to describe the mortality in AYA cancer survivors and its trend across different periods, differences between primary cancer types, age at diagnosis and time since the initial diagnosis. Quantifying the excess mortality in AYA survivors compared to the general population may help in assessing their health needs, forecasting the evolution of the phenomenon, and generating hypotheses on underlying mechanisms.

## 2 Materials and methods

### 2.1 Cohort and variable definitions

An AYA 5-year cancer survivor’s cohort was established in Italy in 2018, pooling together data from 34 population-based Cancer Registries (CRs) which, altogether, cover a population of around 26 million people (43% of the Italian population).

The AYA cohort was established to study the pattern of clinical long-term effects, including mortality, estimate their excess risk, and create an infrastructure to analyse the causes of long-term effects.

The details of the inclusion criteria, the procedures for extracting and preparing the database, and the periods and geographic areas covered by the included CRs have been described elsewhere (15). Briefly, the AYA cohort has a retrospective, incident-based design. Each CR identified patients with a first cancer diagnosis between 15 and 39 years of age among their registered cases, linking them to hospital discharge records (HDRs), and the regional mortality registries. Then the cancer survivors were identified as those alive at least 5 years after the first cancer diagnosis. Primary tumours were grouped by adapting a previously used AYA cancer list (9), based on ICD-O-3 morphology and topography codes (**Supplementary Table 1**). The cohort included 93,291 AYAs diagnosed with cancer between 1976 and 2015, of which 67,692 were AYA cancer survivors (around 72.5% of the total incident cohort).

Since the cause of death was not available for all CRs and, for some CRs, it was not complete for some periods, to analyse the excess mortality risk of AYA cancer survivors, analyses were conducted including the periods of registration for each CR with a satisfactory level of completeness of death cause reporting, arbitrarily set to less than 20% of unknown causes of death. The resulting cohort included 58,387 AYA cancer survivors diagnosed between 1976 and 2013 from 24 CRs (**Supplementary Figure 1**). AYA survivors accrued 427,130 person-years of follow-up, with a median time of 6 years.

The outcomes of interest were all-cause mortality, mortality for cancer (ICD-9 codes, 140-239; ICD-10 codes, C00-D49, including recurrence of primary cancer as second cancers) and for non-neoplastic causes (ICD-9 codes, 001-139, 240-999; ICD-10 codes, A00-B99, D50-Z99). The cancer causes of death occurring in participants with a second cancer registered by the CR were manually checked and classified as probably due to the first cancer and possibly due to the subsequent cancer(s) according to cancer site and type.

All results were presented stratifying by age at diagnosis (15-19, 20-29, 30-39), sex, period of diagnosis (1976-1985, 1986-1995, 1996-2005, 2006-2013), cancer type, the time elapsed since diagnosis in years (5-10, 11-20, 21-30, 31-40), and cancer type (**Supplementary Tables 2**-**4**).

### 2.2 Excess risk analysis

For each AYA cancer survivor, the follow-up started in the fifth year after diagnosis and ended on 31 December 2019 or until death or migration out of the registry area, whichever occurred first.

The standardised mortality ratio (SMR) for the period of diagnosis 1976–2013 with a 95% confidence interval (95% CI) was estimated to compare the relative rate of observed deaths in patients to expected deaths in the general population. The overall excess of death was measured by calculating the Absolute Excess Risks (AERs) per 100,000 person-years by subtracting the expected number of cases from the number of observed, multiplying by 100,000, and finally dividing by person-years at risk. To calculate the expected number of deaths, the person-years (PY) at risk accrued by the AYA cancer survivors during the follow-up were matched, by sex, attained age and area covered by the CR, with the general population mortality rates, taken from the ISTAT website (16).

We estimated SMR for non-cancer causes of death by applying the sex, age, and calendar year-specific percentage of deaths for cancer causes from national statistics to the expected number of deaths. To estimate mortality from cancer and non-cancer causes, deaths with unknown causes were attributed proportionally to cancer and non-cancer deaths according to the proportion of cancer deaths in the period. SMR and AER were also stratified by the period and years since diagnosis for all causes and for cancer and non-cancer causes. Tests for trend comparing SMR and AER across different calendar periods were performed in additive Poisson regression models (17). The Nelson and Alan estimator was used to compute and plot overall all-cause cumulative mortality and stratified by years of diagnosis.

To help interpretation of SMR in survivors after 5 years, we plotted the SMR values of each cancer type according to the 5-year survival observed in a large European cohort. To make the graph meaningful, we plotted only tumour types representing a relatively homogeneous group of cancers, i.e. we excluded the group of “digestive organs”, “female genital tract”, “male genital tract”, “urinary organs”, and “head and neck tumours”. Furthermore, to make the graph less crowded and avoid the influence random fluctuations due to sparse data, we plotted only cancer types with more than 10 observed deaths in study follow-up (4).

The classification of cancer causes as probably due to the first cancer or possibly due to other cancers is presented only as a proportion of all cancer causes in a descriptive analysis, and no excess has been computed.

Analyses were performed using STATA 16.1 software.

### 2.3 Ethics

Ethics approval for this study was obtained from the Institutional Ethics Committee of Fondazione IRCCS Istituto Nazionale dei Tumori, study protocol number INT 0134/17.

## 3 Results

### 3.1 Description of the cohort

In the studied period, 58,387 5-year AYA cancer survivors were followed up for 427,130 person-years; the median follow-up time was 5.7 years beyond the 5^th^ year after diagnosis (maximum follow-up=33 years) and the mean follow-up was 7.3 (**Supplementary Table 5**). By the end of 2019, 4,194 (7.2%) had died, and only 1.6% were lost during the study follow-up.

Sixty-nine per cent of cases were in the age group 30 to 39, while 5.5% were in the age group 15 to 19. The number of included Cancer Registries increased over time; consequently, only 1.8% of cases were diagnosed in the 1976–85 period and 14.2% in the 1986–1995 period, while 41.6% and 42.4% were diagnosed in 1996–2005 and 2006–2013 periods, respectively. Considering the time at risk, the cases diagnosed in the first period accounted for 5.4% of person-years, those in the second period for 31.2%, and those diagnosed in the third and fourth periods for 48.2% and 15.1%, respectively. Most of the person-years (52.8%) were followed up in the period from 5 to 10 years since diagnosis, while only 0.4% of the person years were followed up more than 30 years since diagnosis.

**Figure 1:**
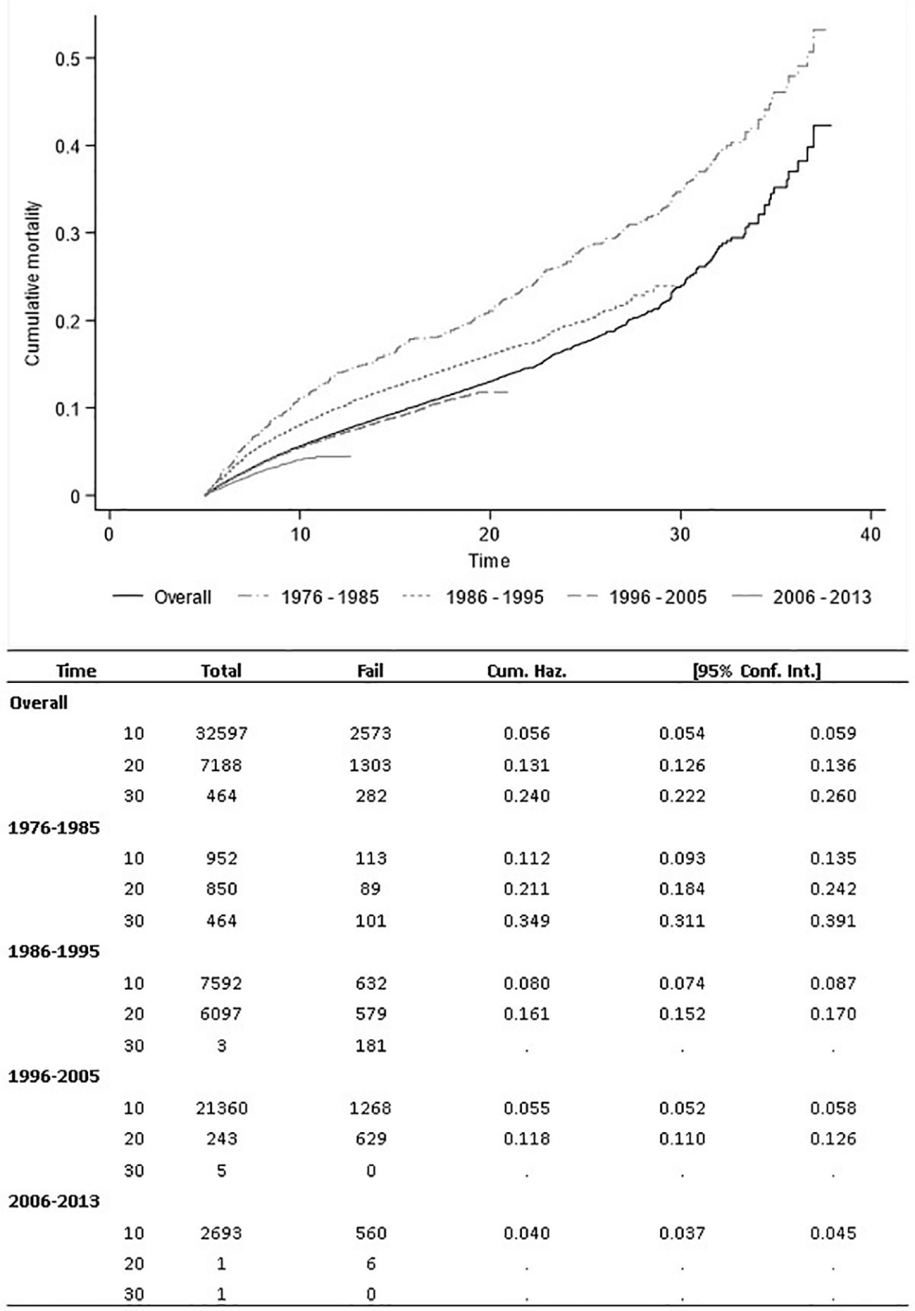
All-causes cumulative mortality.

The most frequent cancer types were breast and thyroid, with 9,823 (16.8%) and 9,163 (15.7%) cases, respectively, corresponding to 66,931 and 59,611 person-years. Other sites with high numbers of cases were lymphomas (8,561, 14.7%), melanomas (6,768, 11.6%), germ cells and trophoblastic tumours (5,639, 9.7%). The high proportion of breast and thyroid cancers, which are more common in females, means that women are 60.1% of the cohort. Moreover, these tumours and melanomas are more common in those aged 30–39 years, which represent 69% of the cases of the cohort. Despite the lower number of included CRs and consequently of cases, about one-half of the deaths (48.6%) occurred in people diagnosed in the first two study periods, i.e. 1976–1985 and 1986-1995. Breast cancers and lymphomas registered about one-half of the deaths that occurred in the cohort, with 36.2% and 13.7%, respectively. The Central Nervous System (CNS) and miscellaneous intracranial and intraspinal neoplasms had the highest proportion of patient deaths at the end of follow-up (25.3%) followed by thymic cancers (22%). Most of the deaths occurred in people diagnosed at 30 to 39 (79.4%) and 5 to 10 years after diagnosis (64.5%) (**Supplementary Table 5**). The all-cause cumulative mortality was 5.6% (95%CI 5.4% - 5.9%), 13.1% (95% CI 12.6% - 13.6%), and 24% (95%CI 22.2% - 26.0%) at 10, 20, and 30 years after diagnosis (**Figure 1**).

In the age group 15-19, the most frequent cancer types were Lymphomas, including reticuloendothelial neoplasms, with 1,123 cases (35.4%) corresponding to 9,238.0 person-years (**Supplementary Table 2**). Also in the 20–29 age group, Lymphomas, including reticuloendothelial neoplasms, were the first cancer with 3,060 (20.4%) cases corresponding to 25,389 person-years. Still, Thyroid and other endocrine gland tumours with 2,673 (17.8%) cases corresponding to 18,675.1 person-years and Germ cell and trophoblastic cancer (excluding gonadal carcinomas) with 2,427 (16.2%) cases corresponding to 18,059.2 person-years were frequent (**Supplementary Table 3**). Breast tumours and Thyroid and other endocrine gland tumours were the most frequent cancer types in the age group 30-39, with 9,123 (22.7%) and 6,077 (15.1%) cases, respectively, corresponding to 62,088.7 and 38,165.9 person-years (**Supplementary Table 4**). Regarding gender, the most frequent cancer types in males were Germ cell and trophoblastic tumours (excluding gonadal carcinomas) and Lymphomas, including reticuloendothelial neoplasms, with 5,346 (22.9%) and 4,569 (19.6%) cases, respectively, corresponding to 39,402.9 and 35,898.5 person-years (**Supplementary Table 6**) while in females Breast tumours and Thyroid and other endocrine gland tumours were the most frequent cancer types with 9,789 (27.9%) and 7,054 (20.1%) cases, respectively, corresponding to 66,687.7 and 46,003.8 person-years (**Supplementary Table 7**).

### 3.2 Excess overall mortality

Compared with the general population, AYA survivors had higher mortality. The all-cause SMR was 7.0 (95%CI 6.8 – 7.2); this SMR corresponds to an AER of 842/100,000 (**Table 1**). In females, the SMR was higher than in males: 9.3 (95%CI 9.0-9.7) and 4.8 (95%CI 4.6-5.1), respectively. The SMR decreased with age at diagnosis, with 8.2 (95%CI 6.8-9.8), 8.1 (95%CI 7.5-8.7), and 6.8 (95%CI 6.6-7.0), in 15-19, 20–29 and 30–39 age groups, respectively; on the contrary, the AER increases with age going from 382, in patients aged 15-19, to 995/100,000, in those aged 30-39. The CNS and miscellaneous intracranial and intraspinal showed the highest SMR, 31.6 (95%CI 28.6-35.0). Among the most frequent cancer types, breast cancer (SMR 16.1, 95%CI 15.3-16.9) showed a higher SMR than the overall cohort, while thyroid and other endocrine glands cancers and germ cells and trophoblastic tumours showed the smallest SMRs (1.5, 95%CI 1.2-1.8 and 1.6, 95%CI 1.4-2.0, respectively). The SMR was higher in the first years after diagnosis (5 to 10 years), 12.8 (95%CI 12.3-13.3), then it decreased, reaching an SMR of 2.2 (95%CI 1.6-3.2) after 30 years since diagnosis. On the contrary, the AER had a U-shaped trend starting with the highest excess, 1106/100,000, decreasing to 548 and 517 in the mid-periods (11 to 30 years from diagnosis), and finally increasing again to 1002/100,000 after 30 years from diagnosis (**Table 1**).

**Table 1:** Observed, expected cases and SMR with 95%CI and AER, by survivor’s characteristics at first diagnosis.

Patient/tumor characteristics	Person	Observed deaths	Expected deaths	SMR (95%CI)	AER
Overall	427,129	4,194	597.5	7.0 (6.8 - 7.2)	842
Age at first diagnosis					
15-19	25,761	112	13.7	8.2 (6.8 - 9.8)	382
20-29	116,032	751	93.1	8.1 (7.5 - 8.7)	567
30-39	285,335	3,331	490.7	6.8 (6.6 - 7.0)	995
Sex					
Male	172,275	1,471	304.9	4.8 (4.6 - 5.1)	677
Female	254,854	2,723	292.6	9.3 (9.0 - 9.7)	954
First primary neoplasm type					
Leukemias, myeloproliferative diseases, and myelodysplastic diseases	18,726	174	24.9	7.0 (6.0 - 8.1)	796
Lymphomas and reticuloendothelial neoplasms	68,074	576	86.4	6.7 (6.1 - 7.2)	719
CNS and miscellaneous intracranial and intraspinal neoplasms	9,688	366	11.6	31.6 (28.6 - 35.0)	3658
Neuroblastoma	389	7	0.5	14.3 (6.8 - 29.9)	1667
Malignant bone tumors	4,336	37	4.7	7.8 (5.7 - 10.8)	745
Soft tissue and other extraosseous sarcomas	17,185	158	25.7	6.1 (5.3 - 7.2)	770
Germ cell and trophoblastic tumors (excluding gonadal carcinomas)	42,000	105	64.8	1.6 (1.4 - 2.0)	96
Malignant melanomas	47,240	304	59.5	5.1 (4.6 - 5.7)	518
Mesotheliomas	183	2	0.3	7.6 (1.9 - 30.6)	925
Thymic tumors	727	25	1.3	19.3 (13 - 28.5)	3258
Eye tumors	269	3	0.3	9.1 (2.9 - 28.3)	1000
Thyroid and other endocrine glands tumors	59,610	104	69.3	1.5 (1.2 - 1.8)	58
Breast tumors	66,931	1518	94.5	16.1 (15.3 - 16.9)	2127
Digestive organs tumors	20,236	278	39.2	7.1 (6.3 - 8.0)	1180
Male genital tract tumors	2,631	12	4.3	2.8 (1.6 - 5.0)	293
Female genital tract tumors	28,625	202	40.3	5.1 (4.4 - 5.8)	565
Urinary tract tumors	23,221	118	42.7	2.8 (2.3 - 3.3)	324
Head and Neck tumors	8,762	134	16.2	8.3 (7.0 - 9.8)	1344
Lung and trachea tumors	3,249	44	5	8.8 (6.5 - 11.8)	1200
Retroperitoneum and peritoneum tumors	87	0	0.1	0.0 (0.0 - 0.0)	114
Other cancers	4,951	27	6.1	4.4 (3.0 - 6.4)	422
Years from diagnosis					
5-10	225,480	2,705	211.2	12.8 (12.3 - 13.3)	1106
11-20	169,890	1,200	269.6	4.5 (4.2 - 4.7)	548
21-30	30,113	259	103.2	2.5 (2.2 - 2.8)	517
31-40	1,646	30	13.5	2.2 (1.6 - 3.2)	1002

SMR, standardized mortality rate; CI, Confidence interval; AER, Absolute Excess Risk.

### 3.3 Distinguishing excess mortality due to cancer and non-cancer causes

SMRs for cancer causes reflect the overall excess mortality with similar patterns across all variables. Of note is that for AYA survivors of breast cancer the excess mortality for cancer causes is particularly high.

SMRs for non-cancer causes are quite homogeneous across all variables and cancer sites. Among cancer types with sufficiently large numbers of deaths to have a precise estimate, only two showed SMRs substantially higher than the average, lymphomas and CNS neoplasms. While the last ones have also the highest SMR for cancer causes, lymphomas have an SMR for cancer causes that is close to the average, if not lower (10.9, 95%CI 9.9-12.0) (**Table 2**). Among the cancer causes (3,429 deaths), those classified as probably attributable to a second cancer, and not to the cancer that led to the inclusion in the cohort, were 11.4%. Among the cancer causes (3,429 deaths), those classified as probably attributable to a second cancer, and not to the cancer that led to the inclusion in the cohort, were 11.4% (390). Looking at specific cancer sites, thyroid cancer has the highest proportion (50%) of cancer deaths due to cancers other than the first cancer, the proportion is high also for germ cell and trophoblastic (44%), male (40%) and female genital (22%), urinary tract (28%) and lymphomas (23%), while breast tumours had one of the lowest proportion (5.4%).The proportion increased dramatically with the increase of the time elapsed since diagnosis, with about 20%, 40% and over 50% after 11-20, 21–30 and 30+ years from diagnosis, respectively (**Table 3**).

**Table 2:** Cohort of AYA cancer survivors.

Patient/tumor characteristics		Cancer deaths				Non cancer deaths			
	Person-years	Observed*	Expected	SMR (95%CI)	AER	Observed*	Expected	SMR (95%CI)	AER
Overall	427,129	3664	300.1	12.2 (11.8 - 12.6)	788	530	297.5	1.8 (1.6 - 1.9)	54
Age at first diagnosis									
15-19	25,761	102	3.4	30.0 (24.4 - 36.1)	382	10	10.3	1.0 (0.5 - 1.7)	-1
20-29	116,032	639	36.7	17.4 (16.1 - 18.8)	519	112	56.4	2.0 (1.6 - 2.4)	48
30-39	285,335	2923	260	11.2 (10.8 - 11.7)	933	408	230.7	1.8 (1.6 - 1.9)	62
Sex									
Male	172,275	1145	116.5	9.8 (9.3 - 10.4)	597	325	188.4	1.7 (1.5 - 1.9)	79
Female	254,854	2519	183.5	13.7 (13.2 - 14.3)	916	205	109.1	1.9 (1.6 - 2.1)	38
First primary neoplasm type									
Leukemias, myeloproliferative diseases, and myelodysplastic diseases	18,726	149	11.1	13.4 (11.4 - 15.7)	736	26	13.8	1.9 (1.2 - 2.7)	65
Lymphomas and reticuloendothelial neoplasms	68,074	422	38.7	10.9 (9.9 - 12)	563	155	47.8	3.2 (2.8 - 3.8)	158
CNS and miscellaneous intracranial and intraspinal neoplasms	9,688	346	4.9	70.2 (63 - 77.8)	3520	20	6.6	3.0 (1.8 - 4.5)	138
Neuroblastoma	389	7	0.2	30 (11.9 - 56.4)	1736	0	0.3	0 (0 - 0)	-66
Malignant bone tumors	4,336	32	2.0	15.9 (10.9 - 22)	682	5	2.7	1.8 (0.6 - 3.8)	52
Soft tissue and other extraosseous sarcomas	17,185	120	12.3	9.7 (8.1 - 11.5)	625	38	13.4	2.8 (2 - 3.8)	143
Germ cell and trophoblastic tumors (excluding gonadal carcinomas)	42,000	65	24.2	2.7 (2.1 - 3.4)	98	39	40.5	1.0 (0.7 - 1.3)	-4
Malignant melanomas	47,240	271	29.5	9.2 (8.1 - 10.3)	511	33	29.9	1.1 (0.8 - 1.5)	6
Mesotheliomas	183	2	0.1	18.6 (1.8 - 53.2)	1029	0	0.2	0 (0 - 0)	-83
Thymic tumors	727	23	0.6	40.2 (25.4 - 58.3)	3072	2	0.7	2.7 (0.3 - 7.9)	175
Eye tumors	269	2	0.2	13.2 (1.2 - 37.9)	685	1	0.2	5.6 (0 - 21.9)	304
Thyroid and other endocrine glands tumors	59,610	72	37.5	1.9 (1.5 - 2.4)	58	31	31.8	1.0 (0.7 - 1.3)	-1
Breast tumors	66,931	1474	59.9	24.6 (23.4 - 25.9)	2113	45	34.6	1.3 (0.9 - 1.7)	16
Digestive organs tumors	20,236	241	19.5	12.3 (10.8 - 14)	1094	38	19.6	1.9 (1.4 - 2.6)	91
Male genital tract tumors	2,631	5	1.6	3.4 (1.2 - 6.9)	147	6	2.7	2.3 (0.8 - 4.4)	127
Female genital tract tumors	28,625	176	25.4	6.9 (5.9 - 8)	526	26	14.9	1.7 (1.1 - 2.5)	39
Urinary tract tumors	23,221	83	19.5	4.3 (3.4 - 5.2)	274	35	23.2	1.5 (1.1 - 2.1)	51
Head and Neck tumors	8,762	115	7.5	15.3 (12.6 - 18.2)	1227	19	8.7	2.2 (1.3 - 3.3)	118
Lung and trachea tumors	3,249	38	2.4	15.8 (11.2 - 21.3)	1087	6	2.6	2.3 (0.8 - 4.4)	103
Retroperitoneum and peritoneum tumors	87	0	0.1	0 (0 - 0)	-60	0	0.1	0 (0 - 0)	-89
Other cancers	4,951	22	3.7	5.8 (3.7 - 8.7)	370	5	2.4	2.1 (0.7 - 4.3)	52
Years from diagnosis									
5-10	225,480	2475	94.3	26.3 (25.2 - 27.3)	1056	231	117	2.0 (1.7 - 2.2)	51
11-20	169,890	980	140.8	7.0 (6.5 - 7.4)	494	220	128.9	1.7 (1.5 - 1.9)	53
21-30	30,113	192	58	3.3 (2.9 - 3.8)	446	66	45.2	1.5 (1.1 - 1.8)	69
31-40	1,646	17	7	2.4 (1.4 - 3.6)	579	13	6.5	2.1 (1.1 - 3.3)	425

*The number includes deaths with unknown cause proportionally attributed to cancer and non-cancer.

Observed and expected death and SMR for cancer and non-cancer causes, by survivor’s characteristics at first diagnosis.

**Table 3:** Number survivors with a second cancer registered and deaths by cancer cause, by survivor’s characteristics at first diagnosis.

Patient/tumor characteristics	Total	Patients with subsequent tumors	Deaths in patients with subsequent tumor	Of which due to subsequent tumors	Percentage of patients dead for subsequent tumors out of total deaths	Percentage of patients dead for subsequent tumors out of cancer deaths
	n	n	n	n	%	%
Overall	58,387	1,765	534	390	9.3	11.4
Age at first diagnosis						
15-19	3,173	53	15	9	8.0	9.9
20-29	15,022	317	72	55	7.3	9.5
30-39	40,192	1,395	447	326	9.8	11.8
Sex						
Male	23,318	604	196	150	10.2	14.2
Female	35,069	1,161	338	240	8.8	10.1
Years of diagnosis						
1976-1985	1,069	163	97	76	22.4	30.4
1986-1995	8,241	551	222	164	11.8	14.5
1996-2005	24,305	730	190	133	7.0	8.4
2006-2013	24,772	321	25	17	3.0	3.6
First primary neoplasm type						
Leukemias, myeloproliferative diseases, and myelodysplastic diseases	2,650	66	27	20	11.5	15.3
Lymphomas and reticuloendothelial neoplasms	8,561	333	123	91	15.8	23.4
CNS and miscellaneous intracranial and intraspinal neoplasms	1,447	19	6	2	0.5	0.6
Neuroblastoma	47	6	0	0	0	0
Malignant bone tumors	520	15	6	4	10.8	13.8
Soft tissue and other extraosseous sarcomas	2,231	60	14	9	5.7	7.8
Germ cell and trophoblastic tumors (excluding gonadal carcinomas)	5,639	113	30	26	24.8	44.1
Malignant melanomas	6,768	156	31	22	7.2	8.7
Mesotheliomas	23	0	0	0	0	0
Thymic tumors	112	5	2	2	8.0	9.1
Eye tumors	33	0	0	0	0	0
Thyroid and other endocrine glands tumors	9,163	229	43	33	31.7	50.0
Breast tumors	9,823	287	120	76	5.0	5.4
Digestive organs tumors	2,569	120	32	22	7.9	10.0
Male genital tract tumors	358	10	2	2	16.7	40.0
Female genital tract tumors	3,271	170	43	36	17.8	22.0
Urinary tract tumors	2,898	83	23	21	17.8	27.6
Head and Neck tumors	1,180	57	23	19	14.2	17.4
Lung and trachea tumors	413	11	7	4	9.1	11.1
Retroperitoneum and peritoneum tumors	13	1	0	0	0	0
Other cancers	668	24	2	1	3.7	5
Years from diagnosis						
5-10	29,194	518	198	130	4.8	5.6
11-20	22,768	807	243	181	15.1	19.9
21-30	6,018	388	91	70	27.0	39.1
31-40	407	52	12	9	30.0	56.3

### 3.4 Time trends

To appreciate any time trend in the SMR it is necessary to stratify by the time elapsed since diagnosis (**Table 4**). The SMR in the period 5 to 10 years since diagnosis decreased from 14.9 (95%CI 12.4-17.8) in people diagnosed from 1976 to 1985 to 11.2 (95%CI 10.3-12.2) in those diagnosed in the period from 2006 to 2013 (p-value for trend <0.01), the corresponding decrease in AER is even more impressive with a three-fold reduction (p-value for trend <0.01). For the other strata, data from recent periods are sparse or not yet observed at all and there are no appreciable trends. Comparing the cumulative mortality according to the calendar period of diagnosis, we observe a decreasing trend in recent periods (**Figure 1**).

**Table 4:** SMR and AER by period and years since diagnosis for all causes and for cancer and non-cancer causes.

All causes																
Years from diagnosis	Years of diagnosis															
	1976-1985				1986-1995				1996-2005				2006-2013			
	Observed	Expected	MR (95%CI)	AER	Observed	Expected	SMR (95%CI)	AER	Observed	Expected	SMR (95%CI)	AER	Observed	Expected	SMR (95%CI)	AER
5-10	119	8	14.9 (12.4 - 17.8)	2198	673	47.4	14.2 (13.2 - 15.3)	1577	1349	105.4	12.8 (12.1 - 13.5)	1066	564	50.4	11.2 (10.3 - 12.2)	802
11-20	92	21.9	4.2 (3.4 - 5.2)	784	558	124.8	4.5 (4.1 - 4.9)	609	548	122.2	4.5 (4.1 - 4.9)	478				
21-30	98	32.6	3.0 (2.5 - 3.7)	874	161	70.6	2.3 (2.0 - 2.7)	400								
31-40	30	13.5	2.2 (1.6 - 3.2)	1004												
Cancer deaths																
Years from diagnosis	Years of diagnosis															
	1976-1985				1986-1995				1996-2005				2006-2013			
	Observed	Expected	SMR (95%CI)	AER	Observed	Expected	SMR (95%CI)	AER	Observed	Expected	SMR (95%CI)	AER	Observed	Expected	SMR (95%CI)	AER
5-10	118	3.3	35.2 (29.1 - 41.8)	2266.4	627	19.7	31.9 (29.4 - 34.4)	1530.1	1216	47.9	25.4 (24 - 26.8)	1000.7	514	23.4	22.0 (20.1 - 23.9)	765.8
11-20	83	11.1	7.5 (6 - 9.2)	808.6	438	65.6	6.7 (6.1 - 7.3)	523.9	459	63.6	7.2 (6.6 - 7.9)	443.4				
21-30	71	18.2	3.9 (3.1 - 4.9)	707.9	121	39.8	3.0 (2.5 - 3.6)	360.1								
31-40	17	7	2.4 (1.4 - 3.6)	579.1												
Non cancer deaths																
Years from diagnosis	Years of diagnosis															
	1976-1985				1986-1995				1996-2005				2006-2013			
	Observed	Expected	SMR (95%CI)	AER	Observed	Expected	SMR (95%CI)	AER	Observed	Expected	SMR (95%CI)	AER	Observed	Expected	SMR (95%CI)	AER
5-10	2	4.7	0.4 (0 - 1.2)	-52.7	46	27.8	1.7 (1.2 - 2.2)	46.6	133	57.5	2.3 (1.9 - 2.7)	64.9	50	27	1.9 (1.4 - 2.4)	35.9
11-20	10	10.8	0.9 (0.4 - 1.6)	-8.5	120	59.2	2.0 (1.7 - 2.4)	85	89	58.6	1.5 (1.2 - 1.9)	34.1				
21-30	27	14.4	1.9 (1.2 - 2.6)	165.6	40	30.7	1.3 (0.9 - 1.7)	39.6								
31-40	13	6.5	2.1 (1.1 - 3.3)	425.1												

SMR, standardized mortality rate; CI, Confidence interval; AER, Absolute Excess Risk.

### 3.5 Time trends of the excess in cancer and non-cancer mortality

Regarding the calendar time trend, the SMR for cancer causes shows a clear decreasing trend in the period 5 to 10 years since diagnosis (p-value for trend <0.01) (**Table 4**). For the deaths due to the first cancer, the trend by period can be distinguished since the beginning of follow-up, while for deaths due to second malignancies the cumulative mortality curves start to diverge later (**Supplementary Figure 2**). In the non-cancer causes, the calendar time trend is appreciable only when excluding the first period, i.e. the years before 1985, in which there is a large proportion of missing cause of deaths (**Supplementary Figure 2**). A non-significant increasing trend for the time elapsed since diagnosis is appreciable only in those diagnosed before 1985 (p-value for trend p=0.09). Similar trends occurred for AER: in the period 5–10 years since diagnosis, for cancer causes there was a decreasing trend (p-value for trend <0.01) but not in the non-cancer causes (p-value for trend p=0.19). It is worth noting that both SMR and AER for non-cancer deaths observed more than 30 years after diagnosis reaches the same magnitude (2.1 and 425/100,000, respectively) as the SMR and AER for cancer causes (2.4 and 579/100,000, respectively).

## 4 Discussion

Our results confirmed an excess risk of death in AYA cancer survivors compared to the general population. Although SMR decreases substantially as the years since diagnosis increase (from 13 to 2), we confirmed that even 30 years after cancer diagnosis, AYA cancer survivors have a 2-fold higher risk of death compared to the general population, i.e. who didn’t have cancer when they were young.

The SMR was higher between 5 and 10 years since diagnosis, and for those diagnosed at a younger age, but AER increased with age at diagnosis. As a rule, SMRs were higher when the mortality in the general population was low and consequently the number of expected cases is very small, as the case for younger age at diagnosis and female sex. On the contrary, the AERs are larger when the mortality in the general population is higher.

Among the cancer types, the excess mortality was higher for CNS neoplasms and breast, while the lowest was for thyroid and germ cell and trophoblastic tumours. CNS tumours are relatively rare in AYAs and very heterogeneous even across age groups: adolescents have a higher proportion of embryonal tumours and a lower proportion of high-grade gliomas than young adults (4). Histological heterogeneity and low incidence make the management of CNS tumours in AYAs difficult. Therefore, despite recent significant advances in neuro-oncology, CNS tumours continue to contribute significantly to AYA mortality (18). Thyroid and germ cell tumours are the AYA cancer with the highest survival and therefore the lowest SMR (**Figure 1**).

Among haematological cancers, SMR was higher for leukaemia than for lymphomas. This is likely the result of intensive treatments with immunosuppressive agents.

In lymphomas and soft tissue sarcomas survivors, the non-cancer causes were notably high despite the all-cause SMRs being close to the average. In breast cancer survivors, the opposite was observed, with low SMR for non-cancer causes and high SMR for cancer causes. Treatment for Hodgkin Lymphoma, the most common lymphoma in AYAs, often includes irradiation to the thyroid region, which increases the risk of thyroid diseases. In addition, evidence has shown that total body irradiation performed in preparation for bone marrow transplantation, which is required in some haematological cancers, results in high risks for gonadal dysfunction, thyroid dysfunction, and adrenal abnormalities, which could all contribute to worse health and non-cancer deaths. Also, for AYA sarcoma survivors, significantly increased mortality for both SMNs and non-cancer causes seemed associated with the initial receipt of chemotherapy or radiotherapy (19). Breast cancer behaviour is more aggressive in AYAs. The peculiar aggressiveness of luminal breast cancer in AYAs might partly be explained by AYA breast cancer genetic features, such as the common enrichment with GATA3 and ARID1A mutations (which predispose to endocrine resistance) and the lower prevalence of PIK3CA mutations (associated with better prognosis). Other contributing factors might be different host characteristics, such as higher basal estrogenic levels, restoration of ovarian function even after chemotherapy, and decreased compliance to hormonal therapy (20).

The SMR for cancer decreased with time elapsing from diagnosis. This could be due to the direct effects of cancer and recurrences, which decrease with time since diagnosis. For the non-cancer deaths, the trend, if any, goes in the opposite direction with increasing SMR when time elapses since diagnosis, most likely due to the long-term impact of cancer and cancer treatment on the general health conditions. In non-cancer mortality, the trend for AER is very strong and the excess mortality reaches almost the same magnitude as that due to cancer-causes 30 years after diagnosis. This observation is consistent with data from the SEER cohort (21).

### 4.1 Comparison with other studies

Our results are consistent with previous results that observed an excess overall mortality between 4 and 10 (13, 22). In all studies, excess cancer-cause mortality is higher in the follow up period closest to the diagnosis. On the contrary, non-cancer mortality increases mostly with age, with absolute risk of dying for non-cancer causes increasing dramatically when the survivors reach the age of fifty, for both cancer survivors and the general population (23). Therefore, the differences in the magnitude of the excess mortality, both relative and absolute, observed in different studies can be mostly explained by the different average lengths of follow-up of the studies and the relative number of person-years observed in the years close to the diagnosis and those observed in a long time (24). Furthermore, the sticky diagnosis bias, leading to overestimation of cancer causes, has been described (25, 26) and could act differently across ages, periods after diagnosis and countries.

In the analyses of calendar time trends, the SMR for cancer causes make evident the improvements in care and diagnosis across the years, consistent with previous studies (27–29). When we compare non-cancer mortality, the differences between AYAs and the general population are smaller than those observed for cancer mortality, and we do not appreciate any calendar time trend in the excess of mortality when measured as relative effect (SMR). Nevertheless, since the mortality in the general population from the ‘70s to the early 2000s decreased markedly, with an increase in life expectancy from 1976 to 2019 of 11 years (23), when we look at the absolute risk, we can appreciate a strong reduction with calendar time for both cancer and non-cancer deaths. These results are partially consistent with those of a large study conducted in the UK on children (<15 years old) cancer survivors that observed a reduction of the AERs for both cancer and non-cancer causes from the ‘60s to the early 2000s (27).

Part of the excess of cancer mortality in our cohort is due to second malignancies. Therefore, our estimate of excess mortality should be interpreted as the sum of the risk of dying from a recurrence of the primary cancer and the risk of dying of a new cancer. Not considering the deaths due to second cancers, which are 390 out of 3,664, the direction and magnitude SMRs and AERs estimates for cancer mortality would not change. Nevertheless, the proportion is higher with increased attained age and time elapsed since diagnosis, as already observed in the UK cohort (27). Thus, for cancer types with lower late mortality, the proportion of deaths due to second malignancies is close to one-half.

**Figure 2:**
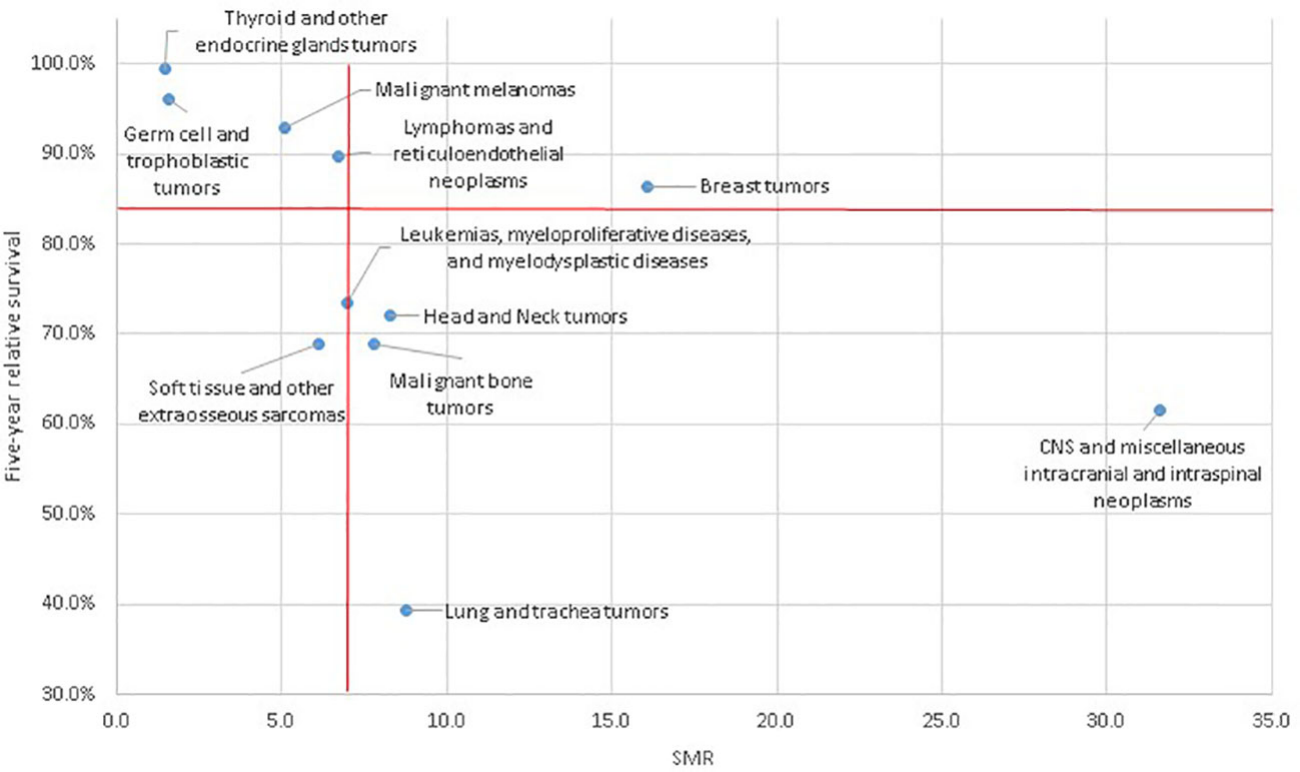
Plot of the 5-year relative survival and late mortality SMR for cancer types.

We showed that the excess mortality was mostly due to the malignancy in the 5–10 years following the diagnosis. Looking at the excess mortality by cancer type, it is worth looking at the long-term excess mortality in light of the 5-year survival. The population included in our study is the result of a selection that occurred in the first years after diagnosis, which differs for different types of cancers. In the case of CNS, a low 5-year survival, i.e. 61.6% (4), continues with a large excess in late mortality with an SMR larger than 30 (**Figure 2**). A symmetrically opposite, but consistent, behaviour can be observed for thyroid cancer and germ cell trophoblastic tumours, which have high survival in the first 5 years and small SMR for late mortality. Interestingly, for these cancers, there is no excess in non-cancer cause late mortality. This is also almost true for melanoma, which has a high 5-year survival, late overall excess mortality slightly lower than the average and almost no excess in non-cancer mortality. Finally, among those that show a similar behavior, lymphomas have both 5-year survival and late mortality close to the overall values. There are also cancers with different behaviors in 5-year survival and late mortality, as for Leukemia, with low 5-year survival and average late mortality. It is worth noting that the late excess for non-cancer causes is on the average for leukemia survivors, while it is large for lymphoma survivors, consistent with previous studies (21, 27, 30). The extreme example of this second behavior is breast cancer, with a high 5-year survival and high SMR for long-term cancer mortality. This finding is consistent with other studies (21). We cannot distinguish whether the death occurred due to a second tumour of the breast or due to relapses of the first cancer. Given the high proportion of BRCA1 and 2 mutation carriers in AYA breast cancers, second cancers are a relatively common event in these women reaching about 10% contralateral breast cancer cumulative incidence in 15 years (31). However, even for non-cancer causes and regardless of the time elapsed since diagnosis, an excess mortality has been reported that is double that of the general population.

### 4.2 Limitations

In Italy, Cancer Registries do not cover the entire population. Furthermore, the existing registries started their activities at different points. Therefore, despite this study including the vast majority of active cancer registries in Italy, the population under study is not the entire Italian population. In particular, very few Cancer Registries contribute to the estimates for the 1976–85 and, to a minor extent, 1986–95 periods. Time trends are surely influenced by the heterogeneity of registries included in different periods compared. Furthermore, despite including virtually all the suitable AYA cancers registered in Italy, the study has no power to investigate trends for specific cancer sites. During the study period, changes in treatment therapy efficacy and toxicity occurred, but their impact on cancer and non-cancer mortality is specific for each cancer type. In this study, we could only appreciate the average result on the population of all cancer patients.

We had to exclude a limited number of registries and short periods of registrations for some registries because the proportion of missing cause-of-death deaths was too high. This further selection had a limited impact on the overall numbers, and all the observed SMRs in the sub-cohort were very close to those observed in the whole cohort. The proportion of missing death causes in the sub-cohort is overall low (6%); nevertheless, it is not evenly distributed, and it is lower for deaths that occurred in patients diagnosed in older periods. If the missing causes are not randomly distributed between cancer and non-cancer, our cause-specific SMRs may be incorrect; the impact of this misclassification could be large on the non-cancer causes SMR in the early periods because the numbers are small, and even a few more observed cases could strongly affect the estimates.

We do not have the detailed cause-specific mortality rates for the reference population for each Cancer Registry in the older period. We could estimate cancer and non-cancer cause-specific mortality rates in the general population through the national statistics reporting the proportion of cancer causes in Italy by geographical macro-area. Thus, we cannot compute detailed cause-specific SMRs for non-cancer causes.

### 4.3 Implications for practice and research

In AYA cancer survivors, the excess mortality is still mostly due to cancer causes up to 30 years after diagnosis. Nevertheless, after 30 years of follow-up, when the survivors reach the age of 45 to 70, and the overall mortality rates in the general population become not negligible, the non-cancer causes equal the cancer causes. Most of these people will be cured when reaching the age when chronic diseases increase their prevalence, second cancer incidence increases, and mortality for non-cancer causes is not a rare event. Thus, particularly for those cancers with a good prognosis, therapeutic choices should be oriented at obtaining good cancer control in the short and medium term but thinking at the long-term survival that will be achievable for most patients, with a balance between the efforts for reducing oncologic risk and primary prevention of other diseases, including second malignancies, must be achieved.

The increase in survival for most cancer sites introduces new needs for AYA cancer survival, not only the issues related to reproductive health but also the healthy ageing issues must be considered in light of their fragility and high life expectancy. The survivorship plan should start from the beginning, when treatment is defined. It would be specific to and the risk factors, including the therapy, intrinsic of the disease and of the patient. The inclusion of non-cancer related specialists in the multidisciplinary tumor board could help obtain a better balance between all the aims of the therapy and survivorship (32, 33).

## 5 Conclusion

All-cause mortality is about 7 times higher in young adult cancer survivors than in the general population. This excess is largely due to mortality due to malignancies, but they also have 80% higher mortality for non-cancer causes. Cancer and non-cancer absolute excess risk become almost similar 30 years after diagnosis. This calls for a survivorship plan in which the prevention of recurrences and other diseases are considered together since the treatment plan though the follow up an after cure is reached.

The choice of the best survivorship plan model for each country calls for understanding the societal, geographic and cultural differences that could be achieved by a comprehensive evaluation of global and local needs. Thus, every country needs to adopt survivorship models that reflect its own health care system (34).

## Data Availability

The raw data supporting the conclusions of this article will be made available by the authors, without undue reservation.

## Ada working group

Manuel Zorzi, Anita Andreano, Paolo Contiero, Gianfranco Manneschi, Fabio Falcini, Marine Castaing, Rosa Angela Filiberti, Cinzia Gasparotti, Claudia Cirilli, Rosalba Amodio, Silvia Iacovacci, Maria Francesca Vitale, Fabrizio Stracci, Maria Adalgisa Gentilini, Rosario Tumino, Simona Carone, Giuseppe Sampietro, Anna Melcarne, Luciana Gatti, Lorenza Boschetti, Mariangela Corti, Magda Rognoni, Enzo Coviello, Maria Teresa Pesce, Giancarlo D’Orsi, Anna Clara Fanetti, Lucia De Lorenzis, Giuseppa Candela, Fabio Savoia, Cristiana Pascucci, Maurizio Castelli, Cinzia Storchi.
