# Supplementary material for: Excess mortality in young cancer survivors compared with the general population in Italy: a retrospective study from the Italian population-based cohort of adolescents and young adult cancer survivors
Source: Front Oncol. 2025 Jul 30;15:1580953. doi: 10.3389/fonc.2025.1580953 (PMC12343270; doi:10.3389/fonc.2025.1580953)
Supplement: Supplementary file 1 [file DataSheet1.docx]

**Supplementary Table 1**. Adolescent and Young Adult Tumor List.

| **Diagnostic group** | **ICD-10 topography** |
| --- | --- |
| Leukemias, myeloproliferative diseases, and myelodysplastic diseases | C91-C95, D45-D47 |
| Lymphomas and reticuloendothelial neoplasms | C81-C86, C88, C90, C96 |
| CNS and miscellaneous intracranial and intraspinal neoplasms | C49, C64, C70-C72, C75 |
| Neuroblastoma | C30, C41, C47-C49, C71, C72, C74-C76 |
| Malignant bone tumors | C31, C40, C41, C49, C71, C72, C75, C76, C80 |
| Soft tissue and other extraosseous sarcomas | C02, C03, C05-C08, C13, C16-C18, C20, C22, C26, C30-C32, C34, C38, C40, C41, C44, C46-C57, C60, C62-C65, C67-C72, C80 |
| Germ cell and trophoblastic tumors (excluding gonadal carcinomas) | C37, C38, C48, C49, C53-C58, C61-C64, C68, C71, C75, C80 |
| Malignant melanomas | C43, C51, C60, C69 |
| Mesotheliomas | C45 |
| Thymic tumors | C37 |
| Eye tumors | C44, C69 |
| Thyroid and other endocrine glands tumors | C73-C75 |
| Breast tumors | C50 |
| Digestive organs tumors | C15-C26 |
| Male genital tract tumors | C60-C63 |
| Female genital tract tumors | C51-C58 |
| Urinary tract tumors | C64-C68, C75, D09, D41 |
| Head and Neck tumors | C00-C14, C30-C32, C44,C76 |
| Lung and trachea tumors | C33, C34, C44 |
| Retroperitoneum and peritoneum tumors | C48 |
| Other cancers | C16-C18, C20, C25, C26, C38,C39,C44, C47, C48, C56, C76, C77, C80 |

**Supplementary Table 2.** Patient characteristics at first diagnosis. Age 15-19.

|  |  |  |  |  |  | **Dead (112)** | | |  |  |
| --- | --- | --- | --- | --- | --- | --- | --- | --- | --- | --- |
|  | **Total** | **Person years** | **Mean (fu)** | **Alive** | **Dead** | **Unknown** | **cancer deaths** | **Non cancer deaths** | **Lost to follow-up** |  |
|  |  |  |  | **n (%)** | **n (%)** | **n (%)** | **n (%)** | **n (%)** | **n (%)** |  |
| **Overall** | 3,173 | 25,761·7 | 8·1 | 3,013 (95·0) | 112 (3·5) | 12 (10·7) | 91 (81·3) | 9 (8·0) | 48 (1·5) |  |
| **Sex** |  |  |  |  |  |  |  |  |  |  |
| Male | 1,665 | 13,586·4 | 8·2 | 1,568 (94·2) | 65 (3·9) | 7 (10·8) | 53 (81·5) | 5 (7·7) | 32 (1·9) |  |
| Female | 1,508 | 12,175·3 | 8·1 | 1,445 (95·8) | 47 (3·1) | 5 (10·6) | 38 (80·8) | 4 (8·5) | 16 (1·1) |  |
| **Years of diagnosis** |  |  |  |  |  |  |  |  |  |  |
| 1976-1985 | 68 | 1,621·4 | 23·8 | 54 (79·4) | 10 (14·7) | 2 (20·0) | 7 (70·0) | 1 (10·0) | 4 (5·9) |  |
| 1986-1995 | 596 | 10,437·4 | 17·5 | 537 (90·1) | 49 (8·2) | 3 (6·1) | 43 (87·8) | 3 (6·1) | 10 (1·7) |  |
| 1996-2005 | 1,172 | 10,229·4 | 8·7 | 1,111 (94·8) | 38 (3·2) | 6 (15·8) | 29 (76·3) | 3 (7·9) | 23 (2·0) |  |
| 2006-2013 | 1,337 | 3,473·5 | 2·6 | 1,311 (98·1) | 15 (1·1) | 1 (6·7) | 12 (80·0) | 2 (13·3) | 11 (0·8) |  |
| **First primary neoplasm type** |  |  |  |  |  |  |  |  |  |  |
| Leukemias, myeloproliferative diseases, and myelodysplastic diseases | 297 | 2,342·8 | 7·9 | 282 (95·0) | 13 (4·4) | 1 (7·7) | 11 (84·6) | 1 (7·7) | 2 (0·7) |  |
| Lymphomas and reticuloendothelial neoplasms | 1,123 | 9,238·0 | 8·2 | 1,066 (94·9) | 43 (3·8) | 7 (16·3) | 31 (72·1) | 5 (11·6) | 14 (1·3) |  |
| CNS and miscellaneous intracranial and intraspinal neoplasms | 157 | 1,482·2 | 9·4 | 139 (88·5) | 16 (10·2) | 1 (6·3) | 15 (93·3) | 0 (0·0) | 2 (1·3) |  |
| Neuroblastoma | 6 | 58·9 | 9·8 | 5 (83·3) | 1 (16·7) | 0 (0·0) | 1 (100) | 0 (0·0) | 0 (0·0) |  |
| Malignant bone tumors | 145 | 1,340·6 | 9·2 | 133 (91·7) | 7 (4·8) | 2 (28·6) | 4 (57·1) | 1 (14·3) | 5 (3·5) |  |
| Soft tissue and other extraosseous sarcomas | 164 | 1,351·5 | 8·2 | 152 (92·7) | 8 (4·9) | 0 (0·0) | 8 (100) | 0 (0·0) | 4 (2·4) |  |
| Germ cell and trophoblastic tumors (excluding gonadal carcinomas) | 348 | 3,041·8 | 8·7 | 335 (96·3) | 6 (1.7) | 0 (0·0) | 5 (83·3) | 1 (16·7) | 7 (2·0) |  |
| Malignant melanomas | 201 | 1,468·3 | 7·3 | 194 (96·5) | 5 (2·5) | 0 (0·0) | 5 (100) | 0 (0·0) | 2 (1·0) |  |
| Mesotheliomas | 1 | 9·5 | 9·5 | 1 (100) | 0 (0·0) | 0 (0·0) | 0 (0·0) | 0 (0·0) | 0 (0·0) |  |
| Thymic tumors | 2 | 8·8 | 4·4 | 1 (50·0) | 0 (0·0) | 0 (0·0) | 0 (0·0) | 0 (0·0) | 1 (50·0) |  |
| Eye tumors | 3 | 47·3 | 15·8 | 3 (100) | 0 (0·0) | 0 (0·0) | 0 (0·0) | 0 (0·0) | 0 (0·0) |  |
| Thyroid and other endocrine glands tumors | 413 | 2,769·8 | 6·7 | 403 (97·6) | 2 (0·5) | 0 (0·0) | 2 (100) | 0 (0·0) | 8 (1·9) |  |
| Breast tumors | 3 | 6·8 | 2·3 | 1 (33·3) | 1 (33·3) | 0 (0·0) | 1 (100) | 0 (0·0) | 1 (33·3) |  |
| Digestive organs tumors | 42 | 285·3 | 6·8 | 37 (88·1) | 4 (9·5) | 0 (0·0) | 4 (100) | 0 (0·0) | 1 (2·4) |  |
| Male genital tract tumors | 14 | 79·9 | 5·7 | 14 (100) | 0 (0·0) | 0 (0·0) | 0 (0·0) | 0 (0·0) | 0 (0·0) |  |
| Female genital tract tumors | 32 | 384·9 | 12·0 | 30 (93·8) | 2 (6·3) | 0 (0·0) | 1 (50·0) | 1 (50·0) | 0 (0·0) |  |
| Urinary tract tumors | 100 | 851·4 | 8·5 | 98 (98·0) | 1 (1·0) | 1 (100) | 0 (0·0) | 0 (0·0) | 1 (1·0) |  |
| Head and Neck tumors | 78 | 565·7 | 7·3 | 75 (96·2) | 3 (3·9) | 0 (0·0) | 3 (100) | 0 (0·0) | 0 (0·0) |  |
| Lung and trachea tumors | 22 | 192·6 | 8·8 | 22 (100) | 0 (0·0) | 0 (0·0) | 0 (0·0) | 0 (0·0) | 0 (0·0) |  |
| Retroperitoneum and peritoneum tumors | 0 | 0·0 | - | - | - | - | - | - | - |  |
| Other cancers | 22 | 235·7 | 10·7 | 22 (100) | 0 (0·0) | 0 (0·0) | 0 (0·0) | 0 (0·0) | 0 (0·0) |  |
| **Years from diagnosis** |  |  |  |  |  |  |  |  |  |  |
| 5-10 | 1,512 | 12,433·0 |  | 1,415 (93·6) | 75 (5·0) | 7 (9·3) | 63 (84·0) | 5 (6·7) | 22 (1·5) |  |
| 11-20 | 1,139 | 10,732·8 |  | 1,097 (96·3) | 25 (2·2) | 3 (12·0) | 20 (80·0) | 2 (8·0) | 17 (1·5) |  |
| 21-30 | 497 | 2,513·0 |  | 476 (95·8) | 12 (2·4) | 2 (16·7) | 8 (66·7) | 2 (16·7) | 9 (1·8) |  |
| 31-40 | 25 | 82·9 |  | 25 (100) | 0 (0·0) | 0 (0·0) | 0 (0·0) | 0 (0·0) | 0 (0·0) |  |

**Supplementary Table 3.** Patient characteristics at first diagnosis. Age 20-29.

|  |  |  |  |  |  | **Dead (751)** | | |  |  |
| --- | --- | --- | --- | --- | --- | --- | --- | --- | --- | --- |
|  | **Total** | **Person years** | **Mean (fu)** | **Alive** | **Dead** | **Unknown** | **cancer deaths** | **Non cancer deaths** | **Lost to follow-up** |  |
|  |  |  |  | **n (%)** | **n (%)** | **n (%)** | **n (%)** | **n (%)** | **n (%)** |  |
| **Overall** | 15,022 | 116,032·5 | 7·7 | 13,947 (92·8) | 751 (5·0) | 69 (9·2) | 580 (77·2) | 102 (13·6) | 324 (2·2) |  |
| **Sex** |  |  |  |  |  |  |  |  |  |  |
| Male | 7,168 | 54,984·9 | 7·7 | 6,648 (92·8) | 342 (4·8) | 35 (10·2) | 240 (70·2) | 67 (19·6) | 178 (2·5) |  |
| Female | 7,854 | 61,047·6 | 7·8 | 7,299 (92·9) | 409 (5·2) | 34 (8·3) | 340 (83·1) | 35 (8·6) | 146 (1·9) |  |
| **Years of diagnosis** |  |  |  |  |  |  |  |  |  |  |
| 1976-1985 | 264 | 5,972·2 | 22·6 | 185 (70·1) | 70 (26·5) | 11 (15·7) | 49 (70·0) | 10 (14·3) | 9 (3·4) |  |
| 1986-1995 | 2,382 | 39,701·9 | 16·7 | 2,043 (85·8) | 286 (12·0) | 25 (8·7) | 214 (74·8) | 47 (16·4) | 53 (2·2) |  |
| 1996-2005 | 6,301 | 54,640·9 | 8·7 | 5,841 (92·7) | 299 (4·8) | 22 (7·4) | 242 (80·9) | 35 (11·7) | 161 (2·6) |  |
| 2006-2013 | 6,075 | 15·717·5 | 2·6 | 5,878 (96·8) | 96 (1·6) | 11 (11·5) | 75 (78·1) | 10 (10·4) | 101 (1·7) |  |
| **First primary neoplasm type** |  |  |  |  |  |  |  |  |  |  |
| Leukemias, myeloproliferative diseases, and myelodysplastic diseases | 746 | 5,376·9 | 7·2 | 686 (91·9) | 45 (6·1) | 7 (15·6) | 33 (73·3) | 5 (11·1) | 15 (2·1) |  |
| Lymphomas and reticuloendothelial neoplasms | 3,060 | 25,389·0 | 8·3 | 2,832 (92·6) | 157 (5·1) | 12 (7·6) | 105 (66·9) | 40 (25·5) | 71 (2·3) |  |
| CNS and miscellaneous intracranial and intraspinal neoplasms | 519 | 3,846·6 | 7·4 | 385 (74·2) | 124 (23·9) | 10 (8·1) | 113 (91·1) | 1 (0·8) | 10 (1·9) |  |
| Neuroblastoma | 19 | 129·4 | 6·8 | 15 (79·0) | 4 (21·1) | 0 (0·0) | 4 (100) | 0 (0·0) | 0 (0·0) |  |
| Malignant bone tumors | 191 | 1,538·0 | 8·1 | 169 (88·5) | 17 (8·9) | 1 (5·9) | 14 (82·4) | 2 (11·8) | 5 (2·6) |  |
| Soft tissue and other extraosseous sarcomas | 639 | 5,198·8 | 8·1 | 586 (91·7) | 36 (5·6) | 2 (5·6) | 26 (72·2) | 8 (22·2) | 17 (2·7) |  |
| Germ cell and trophoblastic tumors (excluding gonadal carcinomas) | 2,427 | 18,059·2 | 7·4 | 2,330 (96·0) | 39 (1·6) | 6 (15·4) | 20 (51·3) | 13 (33·3) | 58 (2·4) |  |
| Malignant melanomas | 1,911 | 14,544·6 | 7·6 | 1,790 (93·7) | 89 (4·7) | 10 (11·2) | 72 (80·9) | 7 (7·9) | 32 (1·7) |  |
| Mesotheliomas | 5 | 54·3 | 10·9 | 5 (100) | 0 (0·0) | 0 (0·0) | 0 (0·0) | 0 (0·0) | 0 (0·0) |  |
| Thymic tumors | 24 | 185·0 | 7·7 | 19 (79·2) | 4 (16·7) | 1 (25·0) | 3 (75·0) | 0 (0·0) | 1 (4·2) |  |
| Eye tumors | 9 | 89·1 | 9·9 | 8 (88·9) | 1 (11·1) | 0 (0·0) | 1 (100) | 0 (0·0) | 0 (0·0) |  |
| Thyroid and other endocrine glands tumors | 2,673 | 18,675·1 | 7·0 | 2,588 (96·8) | 19 (0·7) | 2 (10·5) | 9 (47·4) | 8 (42·1) | 66 (2·5) |  |
| Breast tumors | 697 | 4,835·8 | 6·9 | 571 (81·9) | 115 (16·5) | 5 (4·4) | 108 (93·9) | 2 (1·7) | 11 (1·6) |  |
| Digestive organs tumors | 391 | 3,062·6 | 7·8 | 355 (90·8) | 30 (7·7) | 2 (6·7) | 21 (70·0) | 7 (33·3) | 6 (1·5) |  |
| Male genital tract tumors | 125 | 903·4 | 7·2 | 120 (96·0) | 3 (6·4) | 1 (33·3) | 2 (66·7) | 0 (0·0) | 2 (1·6) |  |
| Female genital tract tumors | 504 | 4,891·1 | 9·7 | 460 (91·3) | 32 (2·7) | 4 (12·5) | 24 (75·0) | 4 (12·5) | 12 (2·4) |  |
| Urinary tract tumors | 557 | 4,984·5 | 8·9 | 533 (95·7) | 15 (2·7) | 3 (20·0) | 9 (60·0) | 3 (20·0) | 9 (1·6) |  |
| Head and Neck tumors | 251 | 2,029·2 | 8·1 | 231 (92·0) | 15 (6·0) | 2 (13·3) | 11 (73·4) | 2 (13·3) | 5 (2·0) |  |
| Lung and trachea tumors | 101 | 928·9 | 9·2 | 96 (95·0) | 3 (3·0) | 1 (33·3) | 2 (66·7) | 0 (0·0) | 2 (2·0) |  |
| Retroperitoneum and peritoneum tumors | 6 | 58·1 | 9·7 | 6 (100) | 0 (0·0) | 0 (0·0) | 0 (0·0) | 0 (0·0) | 0 (0·0) |  |
| Other cancers | 167 | 1,252·7 | 7·5 | 162 (97·0) | 3 (1·8) | 0 (0·0) | 3 (100) | 0 (0·0) | 2 (1·2) |  |
| **Years from diagnosis** |  |  |  |  |  |  |  |  |  |  |
| 5-10 | 7,081 | 58,961·3 |  | 6,421 (90·7) | 474 (6·7) | 37 (7·8) | 395 (83·3) | 42 (8·9) | 186 (2·6) |  |
| 11-20 | 6,054 | 47,812·8 |  | 5,723 (94·5) | 218 (3·6) | 24 (11·0) | 147 (67·4) | 47 (21·6) | 113 (1·9) |  |
| 21-30 | 1,771 | 8,801·2 |  | 1,693 (95·6) | 55 (3·1) | 8 (14·6) | 37 (67·3) | 10 (18·2) | 23 (1·3) |  |
| 31-40 | 116 | 457·2 |  | 110 (94·8) | 4 (3·5) | 0 (0·0) | 1 (25·0) | 3 (75·0) | 2 (1·7) |  |

**Supplementary Table 4**. Patient characteristics at first diagnosis. Age 30-39.

|  |  |  |  |  |  | **Dead (3,331)** | | |  |  |
| --- | --- | --- | --- | --- | --- | --- | --- | --- | --- | --- |
|  | **Total** | **Person years** | **Mean (fu)** | **Alive** | **Dead** | **Unknown** | **cancer deaths** | **Non cancer deaths** | **Lost to follow-up** |  |
|  |  |  |  | **n (%)** | **n (%)** | **n (%)** | **n (%)** | **n (%)** | **n (%)** |  |
| **Overall** | 40,192 | 285,335·6 | 7·1 | 36,307 (90·3) | 3,331 (8·3) | 188 (5·6) | 2,758 (82·8) | 385 (11·6) | 554 (1·4) |  |
| **Sex** |  |  |  |  |  |  |  |  |  |  |
| Male | 14,485 | 103,704·2 | 7·2 | 13,171 (90·9) | 1,064 (7·4) | 72 (6·8) | 762 (71·6) | 230 (21·6) | 250 (1·7) |  |
| Female | 25,707 | 181,631·4 | 7·1 | 23,136 (90·0) | 2,267 (8·8) | 116 (5·1) | 1,996 (88·1) | 155 (6·8) | 304 (1·2) |  |
| **Years of diagnosis** |  |  |  |  |  |  |  |  |  |  |
| 1976-1985 | 737 | 15,530·8 | 21·1 | 449 (60·9) | 259 (35·1) | 29 (11·2) | 194 (74·9) | 36 (13·9) | 29 (3·9) |  |
| 1986-1995 | 5,263 | 83,300·8 | 15·8 | 4,138 (78·6) | 1,057 (20·1) | 37 (3·5) | 874 (82·7) | 146 (13·8) | 68 (1·3) |  |
| 1996-2005 | 16,832 | 140,989·8 | 8·4 | 15,010 (89·2) | 1,560 (9·3) | 85 (5·5) | 1,305 (83·7) | 170 (10·9) | 262 (1·6) |  |
| 2006-2013 | 17,360 | 45,514·2 | 2·6 | 16,710 (96·3) | 455 (2·6) | 37 (8·1) | 385 (84·6) | 33 (7·3) | 195 (1·1) |  |
| **First primary neoplasm type** |  |  |  |  |  |  |  |  |  |  |
| Leukemias, myeloproliferative diseases, and myelodysplastic diseases | 1,607 | 11,006·6 | 6·8 | 1,468 (91·4) | 116 (7·2) | 12 (10·3) | 87 (75·0) | 17 (14·7) | 23 (1·4) |  |
| Lymphomas and reticuloendothelial neoplasms | 4,378 | 33,447·3 | 7·6 | 3,937 (89·9) | 376 (8·6) | 26 (6·9) | 252 (67·0) | 98 (26·1) | 65 (1·5) |  |
| CNS and miscellaneous intracranial and intraspinal neoplasms | 771 | 4,359·6 | 5·7 | 530 (68·7) | 226 (29·3) | 18 (8·0) | 190 (84·1) | 18 (8·0) | 15 (2·0) |  |
| Neuroblastoma | 22 | 201·5 | 9·2 | 20 (90·9) | 2 (9·1) | 0 (0·0) | 2 (100) | 0 (0·0) | 0 (0·0) |  |
| Malignant bone tumors | 184 | 1,457·7 | 7·9 | 168 (91·3) | 13 (7·1) | 0 (0·0) | 11 (84·6) | 2 (15·4) | 3 (1·6) |  |
| Soft tissue and other extraosseous sarcomas | 1,428 | 10,634·7 | 7·4 | 1,292 (90·5) | 114 (8·0) | 3 (2·6) | 82 (71·9) | 29 (25·4) | 22 (1·5) |  |
| Germ cell and trophoblastic tumors (excluding gonadal carcinomas) | 2,864 | 20,899·8 | 7·3 | 2,759 (96·3) | 60 (2·1) | 4 (6·7) | 34 (56·7) | 22 (36·7) | 45 (1·6) |  |
| Malignant melanomas | 4,656 | 31,227·6 | 6·7 | 4,407 (94·7) | 210 (4·5) | 10 (4·8) | 176 (83·8) | 24 (11·4) | 39 (0·8) |  |
| Mesotheliomas | 17 | 120·0 | 7·1 | 15 (88·2) | 2 (11·8) | 0 (0·0) | 2 (100) | 0 (0·0) | 0 (0·0) |  |
| Thymic tumors | 86 | 533·6 | 6·2 | 63 (73·3) | 21 (24·4) | 0 (0·0) | 19 (90·5) | 2 (9·5) | 2 (2·3) |  |
| Eye tumors | 21 | 133·5 | 6·4 | 17 (81·0) | 2 (9·5) | 0 (0·0) | 1 (50·0) | 1 (50·0) | 2 (9·5) |  |
| Thyroid and other endocrine glands tumors | 6,077 | 38,165·9 | 6·3 | 5,912 (97·3) | 83 (1·4) | 7 (8·4) | 55 (66·3) | 21 (25·3) | 82 (1·3) |  |
| Breast tumors | 9,123 | 62,088·7 | 6·8 | 7,621 (83·5) | 1,402 (15·4) | 63 (4·5) | 1,298 (92·6) | 41 (2·9) | 100 (1·1) |  |
| Digestive organs tumors | 2,136 | 16,888·2 | 7·9 | 1,858 (87·0) | 244 (11·4) | 22 (9·0) | 194 (79·5) | 28 (11·5) | 34 (1·6) |  |
| Male genital tract tumors | 219 | 1,647·8 | 7·5 | 207 (94·5) | 9 (4·1) | 0 (0·0) | 3 (33·3) | 6 (66·7) | 3 (1·4) |  |
| Female genital tract tumors | 2,735 | 23,349·4 | 8·5 | 2,524 (91·3) | 168 (6·1) | 9 (5·4) | 139 (82·7) | 20 (11·9) | 43 (1·6) |  |
| Urinary tract tumors | 2,241 | 17,386·1 | 7·8 | 2,103 (93·8) | 102 (4·6) | 6 (5·9) | 67 (65·7) | 29 (28·4) | 36 (1·6) |  |
| Head and Neck tumors | 851 | 6,167·7 | 7·2 | 718 (84·4) | 116 (13·6) | 5 (4·3) | 95 (81·9) | 16 (13·8) | 17 (2·0) |  |
| Lung and trachea tumors | 290 | 2,127·9 | 7·3 | 237 (81·7) | 41 (14·1) | 1 (2·4) | 34 (82·9) | 6 (14·6) | 12 (4·1) |  |
| Retroperitoneum and peritoneum tumors | 7 | 29·4 | 4·2 | 7 (100) | 0 (0·0) | 0 (0·0) | 0 (0·0) | 0 (0·0) | 0 (0·0) |  |
| Other cancers | 479 | 3,462·5 | 7·2 | 444 (92·7) | 24 (5·0) | 2 (8·3) | 17 (70·8) | 5 (20·8) | 11 (2·3) |  |
| **Years from diagnosis** |  |  |  |  |  |  |  |  |  |  |
| 5-10 | 20,601 | 154,086·2 |  | 18,108 (87·9) | 2,156 (10·5) | 121 (5·6) | 1,865 (86·5) | 170 (7·9) | 337 (1·6) |  |
| 11-20 | 15,575 | 111,344·6 |  | 14,451 (92·8) | 957 (6·1) | 58 (6·1) | 744 (77·7) | 155 (16·2) | 167 (1·1) |  |
| 21-30 | 3,750 | 18,798·9 |  | 3,513 (93·7) | 192 (5·1) | 8 (4·2) | 134 (69·8) | 50 (26·0) | 45 (1·2) |  |
| 31-40 | 266 | 1,106·0 |  | 235 (88·4) | 26 (9·8) | 1 (3·8) | 15 (57·7) | 10 (38·5) | 5 (1·9) |  |

**Supplementary Table 5.** Number of survivors, person time in follow up, number of deaths by cause, by survivor’s characteristics at first diagnosis.

|  |  |  |  |  |  | **Dead (4,194)** | | |  |  |
| --- | --- | --- | --- | --- | --- | --- | --- | --- | --- | --- |
|  | **Total** | **Person years** | **Mean (fu)** | **Alive** | **Dead** | **Unknown** | **Cancer deaths** | **Non cancer deaths** | **Lost to follow-up** |  |
|  |  |  |  | **n (%)** | **n (%)** | **n (%)** | **n (%)** | **n (%)** | **n (%)** |  |
| **Overall** | 58,387 | 427,129 | 7.3 | 53,267 (91.2) | 4,194 (7.2) | 269 (6.4) | 3,429 (81.8) | 496 (11.8) | 926 (1.6) |  |
| **Age at first diagnosis** |  |  |  |  |  |  |  |  |  |  |
| 15-19 | 3,173 | 25,761 | 8.1 | 3,013 (95.0) | 112 (3.5) | 12 (10.7) | 91 (81.3) | 9 (8.0) | 48 (1.5) |  |
| 20-29 | 15,022 | 116,032 | 7.7 | 13,947 (92.8) | 751 (5.0) | 69 (9.2) | 580 (77.2) | 102 (13.6) | 324 (2.2) |  |
| 30-39 | 40,192 | 285,335 | 7.1 | 36,307 (90.3) | 3,331 (8.3) | 188 (5.6) | 2,758 (82.8) | 385 (11.6) | 554 (1.4) |  |
| **Sex** |  |  |  |  |  |  |  |  |  |  |
| Male | 23,318 | 172,275 | 7.4 | 21,387 (91.7) | 1,471 (6.3) | 114 (7.8) | 1,055 (71.7) | 302 (20.5) | 460 (2.0) |  |
| Female | 35,069 | 254,854 | 7.3 | 31,880 (90.9) | 2,723 (7.8) | 155 (5.7) | 2,374 (87.2) | 194 (7.1) | 466 (1.3) |  |
| **Years of diagnosis** |  |  |  |  |  |  |  |  |  |  |
| 1976-1985 | 1,069 | 23,124 | 21.7 | 688 (64.4) | 339 (31.7) | 42 (12.4) | 250 (73.8) | 47 (13.8) | 42 (3.9) |  |
| 1986-1995 | 8,241 | 133,440 | 16.2 | 6,718 (81.5) | 1,392 (16.9) | 65 (4.7) | 1,131 (81.2) | 196 (14.1) | 131 (1.6) |  |
| 1996-2005 | 24,305 | 205,860 | 8.5 | 21,962 (90.4) | 1,897 (7.8) | 113 (6.0) | 1,576 (83.0) | 208 (11.0) | 446 (1.8) |  |
| 2006-2013 | 24,772 | 64,705 | 2.6 | 23,899 (96.5) | 566 (2.3) | 49 (8.6) | 472 (83.4) | 45 (8.0) | 307 (1.2) |  |
| **First primary neoplasm type** |  |  |  |  |  |  |  |  |  |  |
| Leukemias, myeloproliferative diseases, and myelodysplastic diseases | 2,650 | 18,726 | 7.1 | 2,436 (91.9) | 174 (6.6) | 20 (11.5) | 131 (7.53) | 23 (13.2) | 40 (1.5) |  |
| Lymphomas and reticuloendothelial neoplasms | 8,561 | 68,074 | 8.0 | 7,835 (91.5) | 576 (6.7) | 45 (7.8) | 388 (67.4) | 143 (24.8) | 150 (1.8) |  |
| CNS and miscellaneous intracranial and intraspinal neoplasms | 1,447 | 9,688 | 6.7 | 1,054 (72.8) | 366 (25.3) | 29 (7.9) | 318 (86.9) | 19 (5.2) | 27 (1.9) |  |
| Neuroblastoma | 47 | 389 | 8.3 | 40 (85.1) | 7 (14.9) | 0 (0.0) | 7 (100) | 0 (0.0) | 0 (0.0) |  |
| Malignant bone tumors | 520 | 4,336 | 8.3 | 470 (90.4) | 37 (7.1) | 3 (8.1) | 29 (78.4) | 5 (13.5) | 13 (2.5) |  |
| Soft tissue and other extraosseous sarcomas | 2,231 | 17,185 | 7.7 | 2,030 (91.0) | 158 (7.1) | 5 (3.2) | 116 (73.4) | 37 (23.4) | 43 (1.9) |  |
| Germ cell and trophoblastic tumors (excluding gonadal carcinomas) | 5,639 | 42,000 | 7.4 | 5,424 (96.2) | 105 (1.9) | 10 (9.5) | 59 (56.2) | 36 (34.3) | 110 (1.9) |  |
| Malignant melanomas | 6,768 | 47,240 | 7.0 | 6,391 (94.4) | 304 (4.5) | 20 (6.6) | 253 (83.2) | 31 (10.2) | 73 (1.1) |  |
| Mesotheliomas | 23 | 183 | 8.0 | 21 (91.3) | 2 (8.7) | 0 (0.0) | 2 (100) | 0 (0.0) | 0 (0.0) |  |
| Thymic tumors | 112 | 727 | 6.5 | 83 (74.1) | 25 (22.3) | 1 (4.0) | 22 (88.0) | 2 (8.0) | 4 (3.6) |  |
| Eye tumors | 33 | 269 | 8.2 | 28 (84.8) | 3 (9.1) | 0 (0.0) | 2 (66.7) | 1 (33.3) | 2 (6.1) |  |
| Thyroid and other endocrine glands tumors | 9,163 | 59,610 | 6.5 | 8,903 (97.2) | 104 (1.1) | 9 (8.7) | 66 (63.5) | 29 (27.8) | 156 (1.7) |  |
| Breast tumors | 9,823 | 66,931 | 6.8 | 8,193 (83.4) | 1,518 (15.5) | 68 (4.5) | 1,407 (92.7) | 43 (2.8) | 112 (1.1) |  |
| Digestive organs tumors | 2,569 | 20,236 | 7.8 | 2,250 (87.6) | 278 (10.8) | 24 (8.6) | 219 (78.8) | 35 (12.6) | 41 (1.6) |  |
| Male genital tract tumors | 358 | 2,631 | 7.4 | 341 (95.2) | 12 (3.4) | 1 (8.3) | 5 (41.7) | 6 (50.0) | 5 (1.4) |  |
| Female genital tract tumors | 3,271 | 28,625 | 8.8 | 3,014 (92.1) | 202 (6.2) | 13 (6.4) | 164 (81.2) | 25 (12.4) | 55 (1.7) |  |
| Urinary tract tumors | 2,898 | 23,221 | 8.0 | 2,734 (94.3) | 118 (4.1) | 10 (8.5) | 76 (64.4) | 32 (27.1) | 46 (1.6) |  |
| Head and Neck tumors | 1,180 | 8,762 | 7.4 | 1,024 (86.8) | 134 (11.4) | 7 (5.2) | 109 (81.4) | 18 (13.4) | 22 (1.8) |  |
| Lung and trachea tumors | 413 | 3,249 | 7.9 | 355 (86.0) | 44 (10.6) | 2 (4.6) | 36 (81.8) | 6 (13.6) | 14 (3.4) |  |
| Retroperitoneum and peritoneum tumors | 13 | 87 | 6.7 | 13 (100) | 0 (0.0) | 0 | 0 | 0 | 0 (0.0) |  |
| Other cancers | 668 | 4,951 | 7.4 | 628 (94.0) | 27 (4.0) | 2 (7.4) | 20 (74.1) | 5 (18.5) | 13 (2.0) |  |
| **Years from diagnosis** |  |  |  |  |  |  |  |  |  |  |
| 5-10 | 29,194 | 225,480 |  | 25,944 (88.9) | 2,705 (9.3) | 165 (6.1) | 2,323 (85.9) | 217 (8.0) | 545 (1.8) |  |
| 11-20 | 22,768 | 169,890 |  | 21,271 (93.4) | 1,200 (5.3) | 85 (7.1) | 911 (75.9) | 204 (17.0) | 297 (1.3) |  |
| 21-30 | 6,018 | 30,113 |  | 5,682 (94.4) | 259 (4.3) | 18 (7.0) | 179 (69.1) | 62 (23.9) | 77 (1.3) |  |
| 31-40 | 407 | 1,646 |  | 370 (90.9) | 30 (7.4) | 1 (3.3) | 16 (53.3) | 13 (43.4) | 7 (1.7) |  |
| **Lenght of follow up after cancer diagnosis (mean, SD)** | 12.8 (6.3) |  |  | 13.0 (6.4) | 10.4 (5.5) |  |  |  | 11.3 (5.8) |  |

**Supplementary Table 6.·**Patient characteristics at first diagnosis. Males.

|  |  |  |  |  |  | **Dead (1,471)** | | | |
| --- | --- | --- | --- | --- | --- | --- | --- | --- | --- |
|  | **Total** | **Person years** | **Mean (fu)** | **Alive** | **Dead** | **Unknown** | **cancer deaths** | **Non cancer deaths** | **Lost to follow-up** |
|  |  |  |  | **n (%)** | **n (%)** | **n (%)** | **n (%)** | **n (%)** | **n (%)** |
| **Overall** | 23,318 | 172,275·4 | 7·4 | 21,387 (91·7) | 1,471 (6·3) | 114 (7·8) | 1,055 (71·7) | 302 (20·5) | 460 (2·0) |
| **Age at first diagnosis** |  |  |  |  |  |  |  |  |  |
| 15-19 | 1,665 | 13,586·4 | 8·2 | 1,568 (94·2) | 65 (3·9) | 7 (10·8) | 53 (81·5) | 5 (7·7) | 32 (1·9) |
| 20-29 | 7,168 | 54,984·9 | 7·7 | 6,648 (92·8) | 342 (4·8) | 35 (10·2) | 240 (70·2) | 67 (19·6) | 178 (2·5) |
| 30-39 | 14,485 | 103,704·2 | 7·2 | 13,171 (90·9) | 1,064 (7·4) | 72 (6·8) | 762 (71·6) | 230 (21·6) | 250 (1·7) |
| **Years of diagnosis** |  |  |  |  |  |  |  |  |  |
| 1976-1985 | 420 | 9,011·9 | 21·5 | 264 (62·9) | 134 (31·9) | 25 (18·7) | 84 (62·7) | 25 (18·7) | 22 (5·2) |
| 1986-1995 | 3,340 | 54,553·1 | 16·3 | 2,755 (82·5) | 522 (15·6) | 29 (5·6) | 370 (70·9) | 123 (23·6) | 63 (1·9) |
| 1996-2005 | 9,661 | 82,761·6 | 8·6 | 8,818 (91·3) | 623 (6·5) | 41 (6·6) | 453 (72·7) | 129 (20·7) | 220 (2·3) |
| 2006-2013 | 9,897 | 25,948·8 | 2·6 | 9,550 (96·5) | 192 (1·9) | 19 (9·9) | 148 (77·1) | 25 (13·0) | 155 (1·6) |
| **First primary neoplasm type** |  |  |  |  |  |  |  |  |  |
| Leukemias, myeloproliferative diseases, and myelodysplastic diseases | 1,475 | 10,494·6 | 7·1 | 1,347 (91·3) | 101 (6·9) | 10 (9·9) | 84 (83·2) | 7 (6·9) | 27 (1·8) |
| Lymphomas and reticuloendothelial neoplasms | 4,569 | 35,898·5 | 7·9 | 4,141 (90·6) | 343 (7·5) | 27 (7·9) | 210 (61·2) | 106 (30·9) | 85 (1·9) |
| CNS and miscellaneous intracranial and intraspinal neoplasms | 801 | 5,276·0 | 6·6 | 566 (70·7) | 219 (27·3) | 16 (7·3) | 192 (87·7) | 11 (5·0) | 16 (2·0) |
| Neuroblastoma | 22 | 178·5 | 8·1 | 18 (81·8) | 4 (18·2) | 0 (0·0) | 4 (100) | 0 (0·0) | 0 (0·0) |
| Malignant bone tumors | 305 | 2,455·6 | 8·1 | 275 (90·2) | 21 (6·9) | 1 (4·7) | 17 (81·0) | 3 (14·3) | 9 (3·0) |
| Soft tissue and other extraosseous sarcomas | 1,097 | 8,254·5 | 7·5 | 979 (89·2) | 88 (8·0) | 4 (4·6) | 57 (64·8) | 27 (30·7) | 30 (2·7) |
| Germ cell and trophoblastic tumors (excluding gonadal carcinomas) | 5,346 | 39,402·9 | 7·9 | 5,138 (96·1) | 102 (1·9) | 10 (9·8) | 57 (55·9) | 35 (34·3) | 106 (2·0) |
| Malignant melanomas | 2,615 | 18,155·4 | 6·9 | 2,424 (92·7) | 158 (6·0) | 12 (7·6) | 124 (78·5) | 22 (13·9) | 33 (1·3) |
| Mesotheliomas | 15 | 128·2 | 8·5 | 13 (86·7) | 2 (13·3) | 0 (0·0) | 2 (100) | 0 (0·0) | 0 (0·0) |
| Thymic tumors | 64 | 479·6 | 7·5 | 51 (79·7) | 10 (15·6) | 0 (0·0) | 10 (100) | 0 (0·0) | 3 (4·7) |
| Eye tumors | 18 | 116·9 | 6·5 | 15 (83·3) | 2 (11·1) | 0 (0·0) | 2 (100) | 0 (0·0) | 1 (5·6) |
| Thyroid and other endocrine glands tumors | 2,109 | 13,607·1 | 6·5 | 2,025 (96·0) | 34 (1·6) | 3 (8·8) | 18 (52·9) | 13 (38·2) | 50 (2·4) |
| Breast tumors | 34 | 243·5 | 7·2 | 25 (73·5) | 9 (26·5) | 1 (11·1) | 8 (88·9) | 0 (0·0) | 112 (1·1) |
| Digestive organs tumors | 1,287 | 10,193·1 | 7·9 | 1,107 (86·0) | 153 (11·9) | 15 (9·8) | 115 (75·2) | 23 (15·0) | 27 (2·1) |
| Male genital tract tumors | 358 | 2,631·2 | 7·4 | 341 (95·2) | 12 (3·4) | 1 (8·3) | 5 (41·7) | 6 (50·0) | 5 (1·4) |
| Female genital tract tumors | - | - | - | - | - | - | - | - | - |
| Urinary tract tumors | 1,970 | 15,434·1 | 7·8 | 1,852 (94·0) | 83 (4·2) | 6 (7·2) | 51 (61·5) | 26 (31·3) | 35 (1·8) |
| Head and Neck tumors | 702 | 5,306·0 | 7·6 | 594 (84·6) | 92 (13·1) | 6 (6·5) | 71 (77·2) | 15 (16·3) | 16 (2·3) |
| Lung and trachea tumors | 206 | 1,702·0 | 8·3 | 174 (84·5) | 23 (11·2) | 1 (4·4) | 17 (73·9) | 5 (21·7) | 9 (4·4) |
| Retroperitoneum and peritoneum tumors | 7 | 61·9 | 8·8 | 7 (100) | 0 (0·0) | 0 | 0 | 0 | 0 (0·0) |
| Other cancers | 318 | 2,255·8 | 7·1 | 295 (92·8) | 15 (4·7) | 1 (6·7) | 11 (73·3) | 3 (20·0) | 8 (2·5) |
| **Years from diagnosis** |  |  |  |  |  |  |  |  |  |
| 5-10 | 11,506 | 90,463·2 |  | 10,333 (89·8) | 896 (7·8) | 61 (6·8) | 701 (78·2) | 134 (15·0) | 277 (2·4) |
| 11-20 | 9,214 | 69,156·8 |  | 8,620 (93·5) | 455 (5·0) | 43 (9·5) | 284 (62·4) | 128 (28·1) | 139 (1·5) |
| 21-30 | 2,439 | 12,051·4 |  | 2,293 (94·0) | 108 (4·4) | 9 (8·3) | 64 (59·3) | 35 (32·4) | 38 (1·6) |
| 31-40 | 159 | 604·0 |  | 141 (88·7) | 12 (7·5) | 1 (8·3) | 6 (50·0) | 5 (41·7) | 6 (3·8) |

**Supplementary Table 7**. Patient characteristics at first diagnosis. Females.

|  |  |  |  |  |  | **Dead (2,723)** | | | |  |
| --- | --- | --- | --- | --- | --- | --- | --- | --- | --- | --- |
|  | **Total** | **Person years** | **Mean (fu)** | **Alive** | **Dead** | **Unknown** | **cancer deaths** | **Non cancer deaths** | **Lost to follow-up** |  |
|  |  |  |  | **n (%)** | **n (%)** | **n (%)** | **n (%)** | **n (%)** | **n (%)** |  |
| **Overall** | 35,069 | 254,854·4 | 7·3 | 31,880 (90·9) | 2,723 (7·8) | 155 (5·7) | 2,374 (87·2) | 194 (7·1) | 466 (1·3) |  |
| **Age at first diagnosis** |  |  |  |  |  |  |  |  |  |  |
| 15-19 | 1,508 | 12,175·4 | 8·1 | 1,445 (95·8) | 47 (3·1) | 5 (10·6) | 38 (80·9) | 4 (8·5) | 16 (1·1) |  |
| 20-29 | 7,854 | 61,047·6 | 7·7 | 7,299 (92·9) | 409 (5·2) | 34 (8·3) | 340 (83·1) | 35 (8·6) | 146 (1·9) |  |
| 30-39 | 25,707 | 181,631·5 | 7·1 | 23,136 (90·0) | 2,267 (8·8) | 116 (5·1) | 1,996 (88·1) | 155 (6·8) | 304 (1·1) |  |
| **Years of diagnosis** |  |  |  |  |  |  |  |  |  |  |
| 1976-1985 | 649 | 14,112·5 | 21·7 | 424 (65·3) | 205 (31·6) | 17 (8·3) | 166 (81·0) | 22 (10·7) | 20 (3·1) |  |
| 1986-1995 | 4,901 | 78,887·1 | 16·1 | 3,963 (80·9) | 870 (17·8) | 36 (4·1) | 761 (87·5) | 73 (8·4) | 68 (1·4) |  |
| 1996-2005 | 14,644 | 123,098·5 | 8·4 | 13,144 (89·8) | 1,274 (8·7) | 72 (5·7) | 1,123 (88·1) | 79 (6·2) | 226 (1·5) |  |
| 2006-2013 | 14,875 | 38,756·4 | 2·6 | 14,349 (96·5) | 374 (2·5) | 30 (8·0) | 324 (86·6) | 20 (5·4) | 152 (1·0) |  |
| **First primary neoplasm type** |  |  |  |  |  |  |  |  |  |  |
| Leukemias, myeloproliferative diseases, and myelodysplastic diseases | 1,175 | 8,231·7 | 7·0 | 1,089 (92·7) | 73 (6·2) | 10 (13·7) | 47 (64·4) | 16 (21·9) | 13 (1·1) |  |
| Lymphomas and reticuloendothelial neoplasms | 3,992 | 32,175·8 | 8·1 | 3,694 (92·5) | 233 (5·8) | 18 (7·7) | 178 (76·4) | 37 (15·9) | 65 (1·6) |  |
| CNS and miscellaneous intracranial and intraspinal neoplasms | 646 | 4,412·4 | 6·8 | 488 (75·5) | 147 (22·8) | 13 (8·8) | 126 (85·7) | 8 (5·4) | 11 (1·7) |  |
| Neuroblastoma | 25 | 211·4 | 8·5 | 22 (88·0) | 3 (12·0) | 0 (0·0) | 3 (100) | 0 (0·0) | 0 (0·0) |  |
| Malignant bone tumors | 215 | 1,880·7 | 8·7 | 195 (90·7) | 16 (7·4) | 2 (12·5) | 12 (75·0) | 2 (12·5) | 4 (1·9) |  |
| Soft tissue and other extraosseous sarcomas | 1,134 | 8,930·5 | 7·9 | 1,051 (92·7) | 70 (6·2) | 1 (1·4) | 59 (84·3) | 10 (14·3) | 13 (1·2) |  |
| Germ cell and trophoblastic tumors (excluding gonadal carcinomas) | 293 | 2,598·0 | 8·9 | 286 (97·6) | 3 (1·0) | 0 (0·0) | 2 (66·7) | 1 (33·3) | 4 (1·4) |  |
| Malignant melanomas | 4,153 | 29,085·1 | 7·0 | 3,967 (95·5) | 146 (3·5) | 8 (5·5) | 129 (88·4) | 9 (6·2) | 40 (1·0) |  |
| Mesotheliomas | 8 | 55·7 | 7·0 | 8 (100) | 0 (0·0) | 0 (0·0) | 0 (0·0) | 0 (0·0) | 0 (0·0) |  |
| Thymic tumors | 48 | 247·9 | 5·2 | 32 (66·7) | 15 (31·3) | 1 (6·7) | 12 (80·0) | 2 (13·3) | 1 (2·1) |  |
| Eye tumors | 15 | 152·9 | 10·2 | 13 (86·7) | 1 (6·7) | 0 (0·0) | 0 (0·0) | 1 (100) | 1 (6·7) |  |
| Thyroid and other endocrine glands tumors | 7,054 | 46,003·8 | 6·5 | 6,878 (97·5) | 70 (1·0) | 6 (8·6) | 48 (68·6) | 16 (22·9) | 106 (1·5) |  |
| Breast tumors | 9,789 | 66,687·7 | 6·8 | 8,168 (83·4) | 1,509 (15·4) | 67 (4·4) | 1,399 (92·7) | 43 (2·9) | 112 (1·1) |  |
| Digestive organs tumors | 1,282 | 10,043·0 | 7·8 | 1,143 (89·2) | 125 (9·8) | 9 (7·2) | 104 (83·2) | 12 (9·6) | 14 (1·1) |  |
| Male genital tract tumors | - | - | - | - | - | - | - | - | - |  |
| Female genital tract tumors | 3,271 | 28,625·5 | 8·8 | 3,014 (92·1) | 202 (6·2) | 13 (6·4) | 164 (81·2) | 25 (12·4) | 55 (1·7) |  |
| Urinary tract tumors | 928 | 7,787·8 | 8·4 | 882 (95·0) | 35 (3·8) | 4 (11·4) | 25 (71·4) | 6 (17·2) | 11 (1·2) |  |
| Head and Neck tumors | 478 | 3,456·7 | 7·2 | 430 (90·0) | 42 (8·8) | 1 (2·4) | 38 (90·5) | 3 (7·1) | 6 (1·2) |  |
| Lung and trachea tumors | 207 | 1,547·4 | 7·5 | 181 (87·4) | 21 (10·1) | 1 (4·8) | 19 (90·5) | 1 (4·8) | 5 (2·4) |  |
| Retroperitoneum and peritoneum tumors | 6 | 25·6 | 4·3 | 6 (100) | 0 (0·0) | 0 | 0 | 0 | 0 (0·0) |  |
| Other cancers | 350 | 2,695·0 | 7·7 | 333 (95·2) | 12 (3·4) | 1 (8·3) | 9 (75·0) | 2 (16·7) | 5 (1·4) |  |
| **Years from diagnosis** |  |  |  |  |  |  |  |  |  |  |
| 5-10 | 17,688 | 135,017·3 |  | 15,611 (88·3) | 1,809 (10·2) | 104 (5·8) | 1,622 (89·7) | 83 (4·6) | 268 (1·5) |  |
| 11-20 | 13,554 | 100,733·3 |  | 12,651 (93·3) | 745 (5·5) | 42 (5·6) | 627 (84·2) | 76 (10·2) | 158 (1·2) |  |
| 21-30 | 3,579 | 18,061·7 |  | 3,389 (94·7) | 151 (4·2) | 9 (6·0) | 115 (76·2) | 27 (17·9) | 39 (1·1) |  |
| 31-40 | 248 | 1,042·1 |  | 229 (92·3) | 18 (7·3) | 0 (0·0) | 10 (55·6) | 8 (44·4) | 1 (0·4) |  |

**Supplementary Figure 1.** Incidence, follow-up and database availability in time by Cancer Registry (lines).


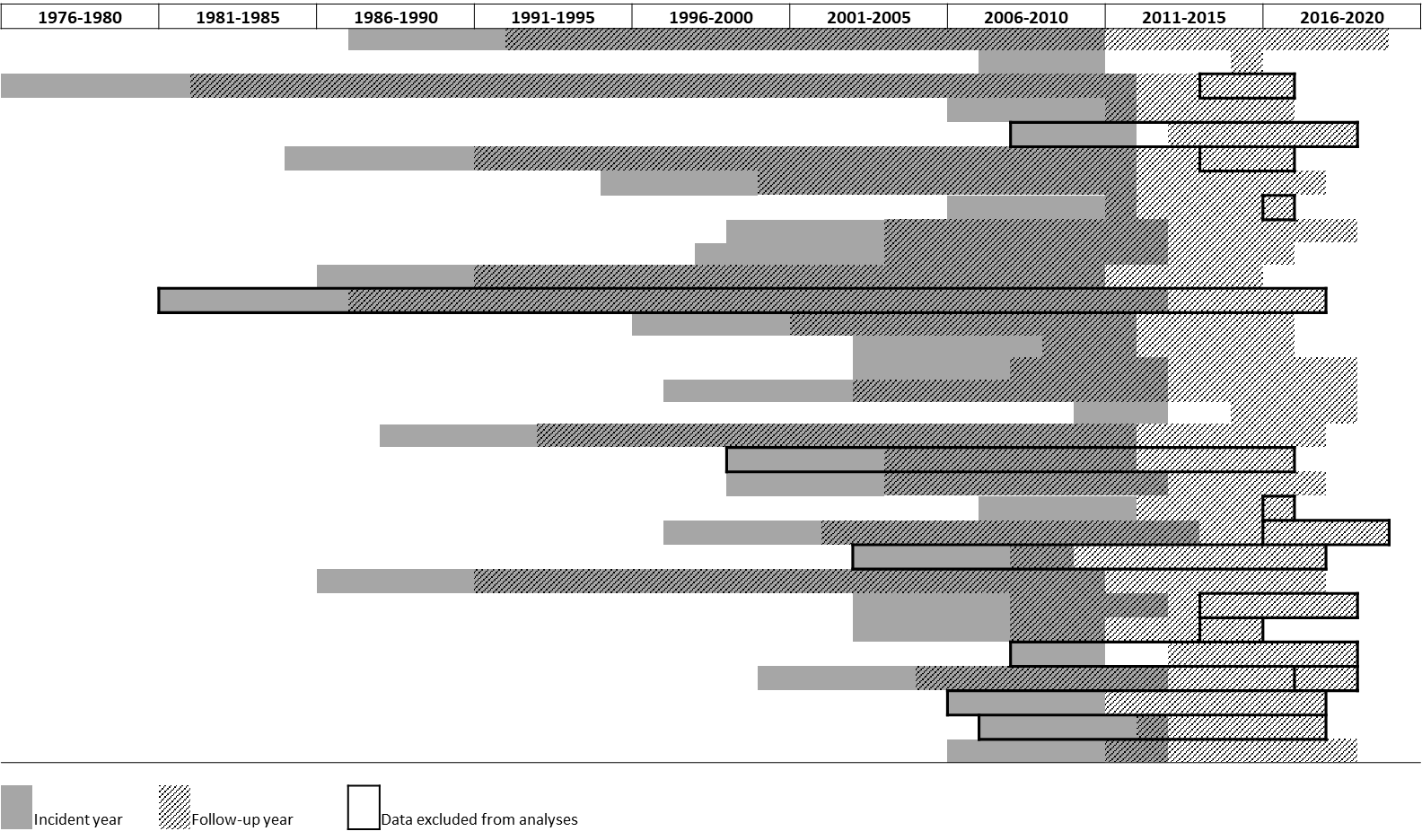


**Supplementary Figure 2.** Cumulative mortality by cause and calendar period of diagnosis.
